# Embryonic transcriptome unravels mechanisms and pathways underlying embryonic development with respect to muscle growth, egg production, and plumage formation in native and broiler chickens

**DOI:** 10.3389/fgene.2022.990849

**Published:** 2022-10-14

**Authors:** M. Kanakachari, R. Ashwini, R. N. Chatterjee, T. K. Bhattacharya

**Affiliations:** ^1^ ICAR-Directorate of Poultry Research, Hyderabad, India; ^2^ EVA.4 Unit, Faculty of Forestry and Wood Sciences, Czech University of Life Sciences Prague, Prague, Czechia

**Keywords:** fast and slow growth chicken, 7th- and 18th-day embryo tissues, microarray, reference gene, quantitative real-time PCR

## Abstract

**Background:** Muscle development, egg production, and plumage colors are different between native and broiler chickens. The study was designed to investigate why improved Aseel (PD4) is colorful, stronger, and grew slowly compared with the control broiler (CB).

**Methods:** A microarray was conducted using the 7th-day embryo (7EB) and 18th-day thigh muscle (18TM) of improved Aseel and broiler, respectively. Also, we have selected 24 *Gallus gallus* candidate reference genes from NCBI, and total RNA was isolated from the broiler, improved Aseel embryo tissues, and their expression profiles were studied by real-time quantitative PCR (qPCR). Furthermore, microarray data were validated with qPCR using improved Aseel and broiler embryo tissues.

**Results:** In the differential transcripts screening, all the transcripts obtained by microarray of slow and fast growth groups were screened by fold change ≥ 1 and false discovery rate (FDR) ≤ 0.05. In total, 8,069 transcripts were differentially expressed between the 7EB and 18TM of PD4 compared to the CB. A further analysis showed that a high number of transcripts are differentially regulated in the 7EB of PD4 (6,896) and fewer transcripts are differentially regulated (1,173) in the 18TM of PD4 compared to the CB. On the 7th^-^ and 18th-day PD4 embryos, 3,890, 3,006, 745, and 428 transcripts were up- and downregulated, respectively. The commonly up- and downregulated transcripts are 91 and 44 between the 7th- and 18th-day of embryos. In addition, the best housekeeping gene was identified. Furthermore, we validated the differentially expressed genes (DEGs) related to muscle growth, myostatin signaling and development, and fatty acid metabolism genes in PD4 and CB embryo tissues by qPCR, and the results correlated with microarray expression data.

**Conclusion:** Our study identified DEGs that regulate the myostatin signaling and differentiation pathway; glycolysis and gluconeogenesis; fatty acid metabolism; Jak-STAT, mTOR, and TGF-β signaling pathways; tryptophan metabolism; and PI3K-Akt signaling pathways in PD4. The results revealed that the gene expression architecture is present in the improved Aseel exhibiting embryo growth that will help improve muscle development, differentiation, egg production, protein synthesis, and plumage formation in PD4 native chickens. Our findings may be used as a model for improving the growth in Aseel as well as optimizing the growth in the broiler.

## Introduction

Animal agriculture production is essential for supplying protein nutrition to the increasing global human population. The broiler chickens are genetically selected with highly improved production efficiency through rapid growth and high feed efficiency compared to improved Aseel native chicken birds. Therefore, understanding mechanisms regulating rapid muscle growth and high feed efficiency between control broiler and improved Aseel may improve the quality of improved Aseel animal production systems (Niemann et al., 2011).

In high and low production efficiency breast muscle phenotypes, male pedigree broiler breeder chickens were used for a global gene expression cDNA microarray study (Kong et al., 2011; Bottje et al., 2012; Bottje and Kong, 2013). Also, RNAseq global gene expression studies have been performed with breast muscle and duodenal tissue in commercial broilers and low and high residual feed intake broilers, respectively (Lee et al., 2015; Zhou et al., 2015). Global gene expression studies mostly showed that production ability could also be related to different cellular mechanisms such as mitochondrial oxidative stress, inflammatory response, protein degradation, stress responses, growth hormone signaling, cell cycle, apoptosis, and fatty acid transportation. A recent transcriptome study reported that differentially expressed genes are enriched in myogenic growth and differentiate on the 6th and 21st day of breast muscle in modern pedigree broiler chickens compared with legacy chicken lines (Davis et al., 2015). A transcriptome analysis was performed with the pectoralis major muscles of slow- and fast-growing chickens (n = 8) to understand the myopathies related to structural changes and molecular pathways using an 8 × 60 K Agilent chicken microarray histological study. For fast-growing breast meat yield, a functional analysis revealed the favoring of metabolic shifts toward alternative catabolic pathways, oxidative stress, inflammation, regeneration, fibrosis processes, cellular defense, and remodeling (Pampouille et al., 2019). A transcriptome profiling analysis was performed in two chicken lines, that is, high (pHu+) and low (pHu-), using an Agilent custom chicken 8 × 60 K microarray. Between these two lines, 1,436 differentially expressed (DE) genes were found, and they were related to biological processes for muscle development and remodeling and carbohydrate and energy metabolism (Beauclercq et al., 2017). A genome-wide association study (GWAS) was conducted to assess body weight in an F2 chicken population, and a microarray expression study was conducted with the liver of high and low-weight chickens. Also, we identified miR-16 as a critical regulator that will suppress chicken embryo cell proliferation and cellular growth. The mutated miR-16 by inserting 54bp showed a significant increase in body weight, bone size, and muscle mass (Jia et al., 2016). The comparative transcriptome analysis of grouper fish muscle in the hybrid and its parents showed up-regulation of genes related to glycolysis, calcium signaling, and troponin pathways that enhanced muscle growth in the hybrid grouper (Sun et al., 2016). In addition, insulin-like growth factor 1 (IGF1) and a cascade of intracellular components (protein kinase B, mTOR, GSK3β, and FoxO) play a significant role in the regulation of skeletal muscle growth during development and regeneration (Schiaffino and Mammucari, 2011).

Muscle growth contains complex network combinations, that is, cell proliferation, differentiation, and metabolism (Fuentes et al., 2013). In mammals, during the embryonic period, the total skeletal muscle fiber number is initiated, and after birth, only muscle hypertrophy occurs (Rowe and Goldspink, 1969; Timson and Dudenhoeffer, 1990; Rehfeldt et al., 2000). In teleosts, hypertrophic and hyperplastic muscle growth can happen simultaneously during the entire life (Stickland, 1983; Weatherley et al., 1988). However, chickens’ total skeletal muscle fiber number is initiated and fixed during the embryonic period (Bhattacharya et al., 2015; Bhattacharya et al., 2019). As a consequence, chicken muscle mass accounts for a better proportion of body weight, and it is an excellent experimental model for studying fundamental growth regulatory mechanisms in vertebrates (Weatherley and Gill, 1985). Therefore, for controlling muscle growth, understanding the mechanisms in chicken is necessary to optimize poultry. In America, during the mid-19th century, dual-purpose chicken, that is, Barred Plymouth Rock (BPR), a foundational or heritage breed of the modern commercial broilers, was developed by crosses with Black Java, Black Cochin, and Dominique breed with alternating white and black bars of feather pigmentation (Lopez et al., 2007; Dorshorst and Ashwell, 2009).

For egg production, multiple gene interactions in various organs regulate energy metabolism, protein synthesis, and storage (Silversides and Villeneuve, 1999). Previous genomic and transcriptomic reports identified genes associated with reproduction traits (Ciacciariello and Gous, 2005). In total, 26 differentially expressed genes (DEGs) were identified in ovaries between pre-laying and egg-laying periods (Kang et al., 2009). The 12 genes identified were related to reproduction regulation pathways such as GnRH, G protein-coupled receptor, calcium-signaling pathways, biosynthesis of steroid hormones, oocyte meiosis, and progesterone-mediated oocyte maturation (Luan et al., 2014). In chickens, nine transcripts related to high egg production were identified in the hypothalamus and the pituitary gland (Shiue et al., 2006). Recently, a comparative transcriptome study was conducted between the hypothalamus and the pituitary gland in Chinese dagu chickens (Wang and Ma, 2019). However, no studies reported how to regulate the genes in embryos for oogenesis and egg development in chickens.

The genetic and developmental foundation of morphological complexity is one of the most significant questions in evolutionary biology, and because avian feathers come in a variety of shapes, they make a great model system for research on the evolution and development of unique morphological features (Losos et al., 2013; Chen B. et al., 2015). Feathers are an excellent model to study the molecular basis of phenotypic variation of an important structure in a single species because they have evolved to have different forms in color, morphology, and mechanical properties not only among different bird species but also in different body regions of an individual bird. The structure and shape of a body feather vary dynamically from the distal end to the proximal end, with the distal end forming before the proximal end. A body feather’s barbs change from being mostly pennaceous at the proximal end to plumulaceous at the distal end (Ng et al., 2015). A great example of exaptation is the feather, which may have originally been developed to regulate body temperature but was later appropriated for show and flight. These and other evolutionary innovations most likely resulted from altered feather-related gene expression patterns. The morphological novelties of feathers are the result of the origin and evolution of plesiomorphic molecular signaling modules (Prum, 2005). A novel technological platform made possible by systems biology research can disclose the molecular expression profiles connected to various morphological processes. The identification of genes linked to changes in feather and scale will be aided by transcriptome investigations and bioinformatic analysis (Ozsolak and Milos, 2011; Chang et al., 2015). A transcriptomic study was conducted on two feather types at different times during their regeneration after plucking and then compared the gene expression patterns in different types of feathers and different portions of a feather and identified morphotype-specific gene expression patterns (Ng et al., 2015). Recently, transcriptome data from yellow and white feather follicles from 7- to 11-week-old F3 chickens were generated to screen for genes involved in the production of pheomelanin particles (Zheng et al., 2020). However, it is still completely unknown what causes feather variance genetically, particularly during embryo development. Understanding the molecular dynamics of embryonic development concerning the process of feather growth can help us better comprehend how different feather shapes have evolved through time.

Thus, the objectives of the present study are to explore mechanisms and pathways underlying embryonic development with respect to muscle growth, egg production, and plumage formation in slow-growing native and fast-growing broiler chickens.

## Materials and methods

### Animals

**FIGURE 1:**
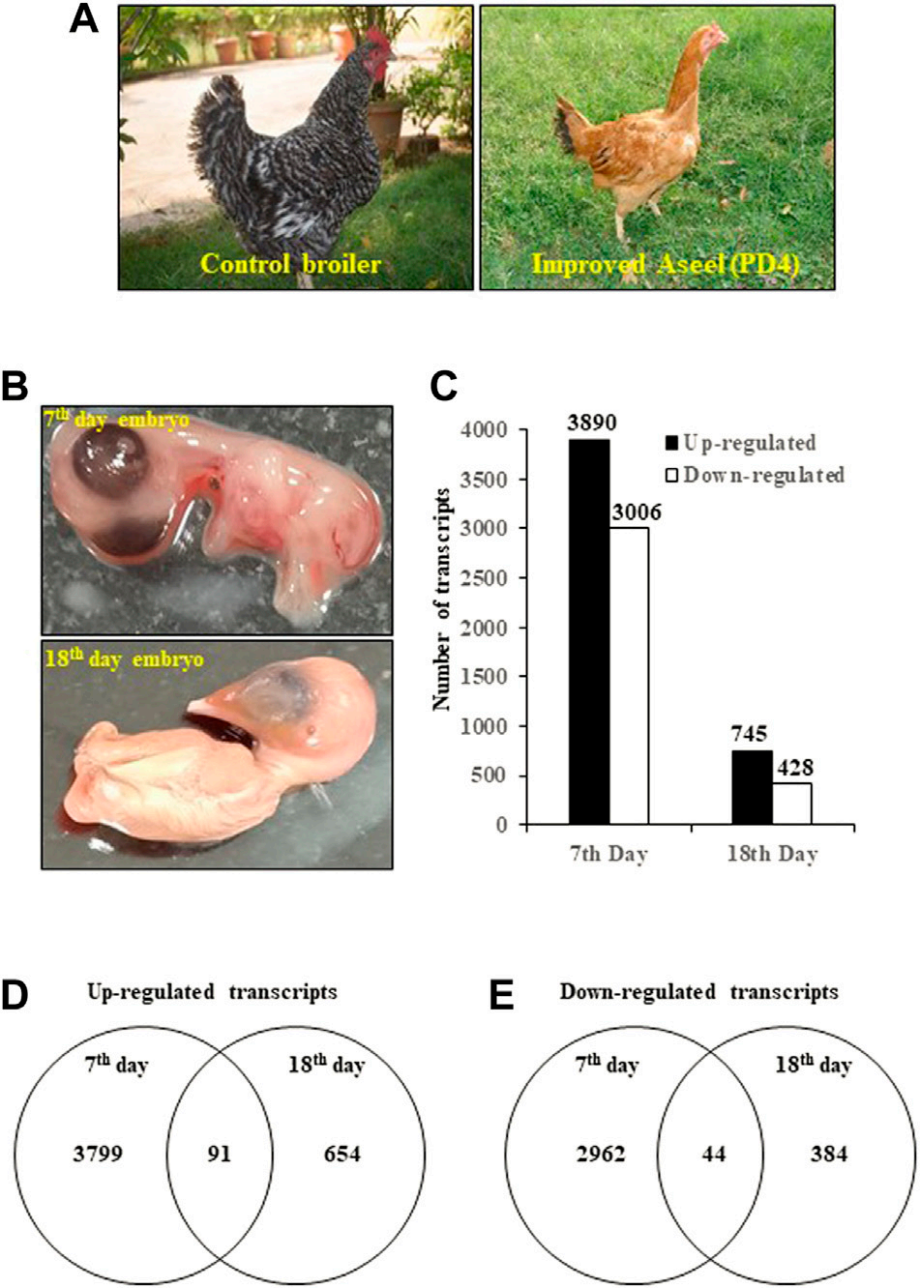
Differentially expressed transcripts/genes (DEGs) in improved Aseel (PD4) during embryo development stages (7th- and 18th-day embryos). **(A)** Fast- (control broiler) and slow-growth (improved Aseel, PD4) birds are used in this study. **(B)** Embryo tissues (complete embryos from the 7th day and thigh muscles from the 18th day) are used for the transcriptome analysis. **(C)** Number of up- and downregulated transcripts (DETs) (*p* value ≤0.05 and fold change ≥1) in Aseel as compared to control broiler samples. **(D)** and **(E)** Number of common and differentially expressed transcripts among the initial (7EB) and developed (18TM) embryo stages.

The study was conducted on the fast-growing broiler pure line (developed from the synthetic population) and improved Aseel (PD4) chicken lines maintained at the institute farm, ICAR-Directorate of Poultry Research, Hyderabad, India (Figure 1A). The improved Aseel (PD-4) has been developed from the Indian native Aseel breed of chicken by imposing selection for body weights at 8 weeks of age for the last 10 generations. The body weight of these birds at 8 weeks during the S-10 generation was 551.0 ± 3.60 g. The control broiler birds are random-bred broilers, and there is no selection imposed on this population. The body weight of the control broiler line at 6 weeks was 951.0 ± 1.20 g. The birds of both populations were maintained under an intensive management system. A total of 60 fertile eggs were kept for hatching (30 for each group) in the incubator (Global Incubators, Hyderabad, India) at 100.3°F temperature and 79.2°F humidity. After the 7 and 18 days of incubation, eggs were harvested (15 for each group), and embryos were collected and stored at −80°C up to total RNA isolation.

### RNA extraction and evaluation

For RNA isolation, the complete embryo from the 7th-day and thigh muscle from the 18th-day embryo were used from the control broiler and PD4 lines. The tissue samples were collected from three independent embryos during each time point to isolate RNA and consider each replicate as one biological replicate during each period. Total RNA was isolated using Trizin RNA extraction reagent (GCC Biotech (India) Pvt. Ltd.), according to the manufacturer’s protocol, and RNA was puried by DNase treatment (DNase I solution, HiMedia, India) to remove a trace amount of DNA. The purity and quantity were monitored on 1.2% denatured agarose gels and the NanoDrop 1,000 Spectrophotometer (Thermo Scientic, United States). The quality of total RNA was assessed by checking 200–300 ng of total RNA on an RNA nano chip using an Agilent Bioanalyzer 2100 (Agilent Technologies, United States), according to the manufacturer’s instructions.

### RNA labeling, amplification, and hybridization

The Agilent Quick Amp Kit (Part number: 5190–0442) was used for sample labeling. In addition, 500 ng of total RNA was reverse transcribed using an oligo dT primer tagged to a T7 promoter sequence, and in the same reaction, the cDNA thus obtained was converted to double-stranded cDNA. Labeled cRNA preparation and hybridization on GeneChip and scanning were done following Affymetrix protocols (http://www.affymetrix.com). In the *in vitro* transcription step, the cDNA was converted to cRNA using the T7 RNA polymerase enzyme, Cy3 dye was added to the reaction mix and incorporated into the newly synthesized strands, and the obtained cRNA was cleaned up using Qiagen RNeasy columns (Qiagen, Cat No: 74106). The concentration and amount of dye incorporated were determined using a NanoDrop 1,000 Spectrophotometer (Thermo Fisher Scientic, United States). The QC-passed samples for specific activities were taken for hybridization. Then 600 ng of labeled cRNA was hybridized on the array using the Gene Expression Hybridization Kit (Part Number 5190–0404; Agilent Technologies, United States) in Sure Hybridization Chambers (Agilent technologies, United States) at 65°C for 16 h. Agilent Gene Expression Wash Buffers (Part No: 5188–5327) were used for washing the hybridized slides, and then the slides were scanned on a G2505C scanner (Agilent Technologies, United States).

### Microarray data analysis

After scanning, DAT, CEL, CHP, XML, and JPEG image were generated for each array with Feature Extraction Software (Version-10.7, Agilent Technologies, United States). The CELles containing estimated probe intensity values were further analyzed with GeneSpring GX-11.0 software (Agilent Technologies, United States). Normalization of the data was performed in GeneSpring GX using the 75th percentile shift, and this normalization takes each column in an experiment independently and computes the *n* ^th^ percentile of the expression values for this array across all spots; fold change was calculated concerning specific control samples. Genes were up- and down-regulated showing one-fold and above within the samples concerning the control sample were identified, and for the replicates, a Student’s *t*-test *p*-value was calculated. The expression data obtained have been submitted to the Gene Expression Omnibus database (GEO, http://www.ncbi.nlm.nih.gov/geo) at the National Center for Biotechnology Information with the accession numbers GSE62443-GSE62445.

### Hierarchical clustering analysis

The differentially expressed genes between the 7th^-^ and 18th-day of the embryo were subjected to a hierarchical cluster analysis using the Cluster 3.0 program (Eisen et al., 1998). We imported the matrix with as many columns as stages and rows as genes, where each cell contains the log2 transformed fold change value for the gene and individual into the Cluster 3.0 program, normalizing on rows. To demonstrate the expression pattern and tree diagram of DETs, we applied rows and columns to the cluster 3.0 software and carried out hierarchical clustering using the complete linkage approach with the Euclidean distance. The resulting dendrogram was then exported as an image file.

### Functional characterization

Biological data and analysis tools are combined in the Database for Annotation Visualization and Integrated Discovery (DAVID; https://david.ncifcrf.gov/), which provides systematic functional annotation for a large number of genes and proteins. GO annotation linked with biological processes, molecular function, and KEGG pathway enrichment analyses were carried out using the online DAVID tool version 6.7 (11,12) to examine the possible functions of discovered DEGs (Huang et al., 2009a,b). The FDR *p*-value of < 0.05 and fold change of > 1 were considered to be significant.

### Pathway analyses

Ingenuity Pathway Analysis (IPA; Qiagen, Valencia, CA; http://www.ingenuity.com) software was used for functional annotation, canonical pathway analysis, upstream analysis, and network discovery. The chicken DEGs data set functionalities are primarily based on mammalian biological mechanisms because IPA is based on human bioinformatics. We have attempted to draw possible conclusions based on avian-based literature, but biomedical research biases the functional annotations toward human disease.

### Selection of candidate reference genes

A total of 24 candidate reference genes were chosen based on their previous use/study in chicken or other avian species; the sequences were downloaded from NCBI (https://www.ncbi.nlm.nih.gov/), and the CDS (coding DNA sequence) region was identified by using the ExPASy translation tool (https://web.expasy.org/translate/) (Supplementary Table S1).

### Real-time quantitative PCR analysis

Microarray expression data were validated using two-step real-time quantitative PCR (qPCR) for specific confirmation of the differentially expressed genes. First-strand cDNA was synthesized from 2 µg of total RNA using the Thermo Fisher Scientific Verso cDNA Synthesis Kit (Thermo Scientific, United States). Gene speciac qPCR primers were designed for 24 housekeeping genes and 83 DEGs using PrimerQuest software (http://eu.idtdna.com; Supplementary Table S1; Table 1). The qPCR was performed using the BrightGreen 2X qPCR MasterMix-No Dye (Applied Biological Materials Inc. Canada) in the Insta Q96™ Real-Time Machine (HiMedia Laboratories, India) detection system. The PCR was performed under the following program: 5 min at 95°C followed by 40 cycles of amplication with 15 s of denaturation at 95°C and 60 s of annealing/extension at 60°C. A total of three biological replicates were used. A melt curve analysis was performed to check the special city of the amplied products. The 2^−ΔΔCt^ calculated the relative expression level of each gene, and Transferrin (TFRC; Accession No: X55348.1) from *G. gallus* was used as a housekeeping gene to normalize the amount of template cDNA added in each reaction.

**TABLE 1:** List of primers used for qPCR to validate microarray data

S. No	Gene Name	Accession No.	Primer Sequence	Tm	GC%	Amplicon Size (bp)
1	Destrobrevin alpha (D alpha)	CR733292.1	5′-CAA​CCC​TTT​GTG​GAG​GAA​AGA-3′	62	47.6	114
			5′-GAA​CCT​CCC​GCA​GAA​ACA​A-3′	62	52.6	
2	Uncharacterized protein (UP5)	ES605836.1	5′-GAA​CCA​AAT​GCT​GGC​AGA​AG-3′	62	50	112
			5′-AAA​TAC​TCT​CTG​GGT​GAA​CAG​G-3′	62	45.5	
3	Toll-interacting protein (TOLLIP)	NM_001006471.1	5′-GTG​TAA​CGA​AGA​GGA​CCT​GAA​A-3′	62	45.5	95
			5′-TGT​TCC​CTC​TCT​GAG​CTT​CTA-3′	62	47.6	
4	Asw	CN225783.1	5′-GGC​AAC​ACG​TGA​AAT​CCA​TTC-3′	62	47.6	119
			5′-GCG​CAC​GTC​TCT​GTA​TTC​T-3′	62	52.6	
5	Chain A, fibrinogen alpha subunit (Chain A FAS)	BX935039.1	5′-TGA​CGA​CAC​AGA​CCA​GAA​TTA​C-3′	62	45.5	106
			5′-GGT​TTC​CAC​AAT​TAC​CCG​ATT​G-3′	62	45.5	
6	Hypothetical protein (HP29)	AW198329.1	5′-CCC​AGA​TGA​CAG​AAG​AAC​AAA​TAA​AG-3′	62	38.5	106
			5′-CCC​TCT​TCT​CCA​AAG​CAT​GTA​T-3′	62	45.5	
7	Fibrinogen gamma chain precursor (FGCP)	BG642009.1	5′-CTG​GTC​ACC​TCA​ATG​GAC​AAT​A-3′	62	45.5	106
			5′-CAT​CGG​TCA​CGC​CAT​GTT-3′	62	55.6	
8	Apolipoprotein B precursor (ALPBP)	NM_001044633.1	5′-CTT​GAG​GCC​AAC​TCC​AAA​GTA-3′	62	47.6	102
			5′-GTG​CTC​CCA​GAC​TGC​ATA​AA-3′	62	50	
9	Maestro heat-like repeat–containing protein family member 2B (MHCRCP2B)	CR406681.1	5′-CTG​GAA​CAC​ACC​ACA​GAC​TT-3′	62	50	130
			5′-CCC​GAT​AGA​TGT​CCT​TTC​CAT​AC-3′	62	47.8	
10	Activin A receptor, type IB (AARIB)	XM_001231300	5′-GCA​CGG​ATC​TCT​CTT​TGA​CTA​C-3′	62	50	120
			5′-TGA​GTA​CCC​ACG​ATC​TCC​AT-3′	62	50	
11	cAMP responsive element modulator (CREM)	NM_204387	5′-CAA​GAG​AGA​GCT​GCG​ACT​TAT​G-3′	62	50	102
			5′-AGC​ACA​GCC​ACA​CGA​TTT-3′	62	50	
12	Caveolin 1, caveolae protein, 22kDa (CAV1)	NM_001105664	5′-CAT​TCC​CAT​GGC​ACT​CAT​CT-3′	62	50	106
			5′-GCA​CTG​GAT​C′CAA​TCA​GGT​AG-3′	62	50	
13	Caveolin 2 (CAV2)	NM_001007086	5′-TGC​TGT​ACA​AGC​TGC​TGA​G-3′	62	52.6	140
			5′-CAC​TGA​AGG​CAA​GAC​CAT​GA-3′	62	50	
14	Follistatin-like 1 (FSTL1)	NM_204638	5′-CGA​TGA​CAT​GTG​AAG​GGA​AGA-3′	62	47.6	105
			5′-TCT​GCA​GCT​CCT​GAA​CAT​ATC-3′	62	47.6	
15	WAP, follistatin/kazal, immunoglobulin, kunitz, and netrin domain containing 1 (WAPFK)	NP9672441	5′-GAG​GGC​AAC​AAC​AAC​AAC​TTC	62	47.6	109
			5′-TCA​GCA​CCA​TCT​TGC​TCT​TC-3′	62	50	
16	Glucose-6-phosphate isomerase (GPI)	NM_001006128	5′-CAC​TTC​TGC​CCT​ATG​ACC​AAT​A-3′	62	45.5	110
			5′-GTA​GTC​CAC​ACG​AGA​TCC​TTT​C-3′	62	50	
17	Solute carrier family 2 (facilitated glucose transporter), member 3 (SLC2A3)	NM_205511	5′-GTG​GTA​CAC​AGG​ATG​TAT​CTC​AAG-3′	62	45.8	112
			5′-CGA​TAG​TTT​GGA​GAG​CGG​AAT​AG-3′	62	47.8	
18	Heat shock 60kDa protein 1 (chaperonin) (HSPD1), nuclear gene encoding mitochondrial protein	NM_001012916	5′-GGT​GAG​AAG​GCT​CAG​ATT​GAA-3′	62	47.6	122
			5′-GCT​ACT​CCG​TCA​GAT​AGT​TTG​G-3′	62	50	
19	Heat shock 70kDa protein 5 (glucose-regulated protein, 78kDa) (HSPA5)	NM_205491	5′-TGA​GAC​AGT​TGG​AGG​TGT​AAT​G-3′	62	45.5	103
			5′-AGT​GGG​CTG​ATT​GTC​AGA​AG-3′	62	50	
20	Heat shock 70kDa protein 8 (HSPA8)	NM_205003	5′-AGT​TTG​AGC​TGA​CCG​GTA​TTC-3′	62	47.6	122
			5′-CTC​CTT​GCC​AGT​GCT​CTT​ATC-3′	62	52.4	
21	Heat shock factor binding protein 1 (HSBP1)	NM_001112809	5′-ATG​CAG​GAC​AAA​TTT​CAA​ACC​A-3′	62	36.4	118
			5′-CTA​CTC​CCG​CTT​GTG​TCA​TC-3′	62	55	
22	Heat shock transcription factor 1 (HSTF 1)	BM440477	5′-GCA​GCA​GAA​GGT​GGT​CAA​TA-3′	62	50	146
			5′-AGT​ACT​GGC​GGC​TGT​ATT​TC-3′	62	50	
23	Partial mRNA for heat shock protein 70 (hsp70 gene)	AJ301880	5′-CCC​AGT​AAG​TGC​GGG​TCA​TAA-3′	64	52.4	85
			5′-CGC​TCC​GCC​AGT​CAC​TT-3′	64	64.7	
24	Homeobox C9 (HBC9)	BX950823	5′-AGA​TGT​CCG​TAC​ACA​AAG​TAT​CA-3′	62	39.1	105
			5′-GTT​TAG​GAC​TCG​GGC​TAC​TTC-3′	62	52.4	
25	Insulin-like growth factor 1 receptor (IGF1R)	NM_205032	5′-TGT​GTA​CGT​TCC​AGA​CGA​ATG-3′	62	47.6	104
			5′-CCT​TGG​CTA​TTC​CCT​CAT​ACA​C-3′	62	50	
26	Insulin-like growth factor binding protein 1 (IGFBP1)	NM_001001294	5′-CAG​GAC​CAG​ATG​CTG​AAC​TAT​C-3′	62	50	134
			5′-CCC​TGT​TCT​TTC​CAT​TTC​TTG​TG-3′	62	43.5	
27	Mitogen-activated protein kinase 8 interacting protein 3 (MAPK8IP3)	XM_424591	5′-GTG​ATG​ACA​ACA​GCG​ACA​AAT​C-3′	62	45.5	118
			5′-CCA​GGC​ACA​GAG​ACA​AAG​AA-3′	62	50	
28	Mitogen-activated protein kinase kinase kinase 4 (MAPKKK4)	CR523470	5′-AGT​GGA​TGA​ACT​ACG​TGC​TAA​C-3′	62	45.5	120
			5′-CCG​GGA​GAG​CCG​AAA​TAA​AT-3′	62	50	
29	Mitogen-activated protein kinase-activated protein kinase 3 (MAPKAPK3)	XM_414262	5′-CTG​AAG​ACT​GAC​CCA​ACA​GAG-3′	62	52.4	139
			5′-CTT​CAT​CCC​AGT​GGT​CCT​TAT​C-3′	62	50	
30	Myozenin 2 (MZ2)	BX930590	5′-GAA​ACA​ACA​AGC​ATC​AGC​CAT​TA-3′	62	39.1	121
			5′-GCT​GAG​TGT​TGA​TAG​TTC​CTC​TAC-3′	62	45.8	
31	Angiotensin II receptor, type 1 (AGTR1)	NM_205157	5′-TTC​CTG​GAT​TCC​TCA​TCA​AGT​G-3′	62	45.5	103
			5′-GGG​CAT​AGC​TGT​ATC​CAC​AAT​A-3′	62	45.5	
32	Angiotensin II receptor–associated protein (AGTRAP)	BX930324	5′-CTT​CAA​CAT​AGG​TCT​CAA​CCG​T-3′	62	45.5	106
			5′-CTG​AGC​TGC​CTT​GCT​TGA-3′	62	55.6	
33	CD9 molecule (CD9)	NM_204762	5′-TAC​TAC​AAT​GCC​ATG​CCC​TAA​A-3′	62	40.9	134
			5′-TAG​CAC​AGC​AAA​GAA​CCA​TAC​T-3′	62	40.9	
34	Dickkopf homolog 2 (DKK2)	XM_420494	5′-CGC​AAC​AAG​AAG​AAC​AGT​CAT​TAT-3′	62	37.5	105
			5′-GGG​ATC​ACC​TTC​ATG​TCC​TTT​A-3′	62	45.5	
35	Glycoprotein M6A (GPM6A)	NM_001012579	5′-GGA​TCT​TCG​CCA​GTA​TGG​TAT​T-3′	62	45.5	97
			5′-TAG​CTC​ATT​CGA​GTC​ACA​CAT​C-3′	62	45.5	
36	Glycoprotein M6B (GPM6B)	NM_001012545	5′-GAA​CAT​CTG​CAA​CAC​GAA​TGA​G-3′	62	45.5	124
			5′-GGC​CCA​GTT​AGA​AGA​CAG​TAT​C-3′	62	50	
37	Janus kinase 1 (JAK1)	NM_204870	5′-CAA​GGA​ACT​AGC​TGA​CCT​GAT​G-3′	62	50	98
			5′-CCT​CCA​GTT​TGT​TGA​TGT​CTC​T-3′	62	45.5	
38	Janus kinase 2 (JAK2)	NM_001030538	5′-GAT​GGA​TGC​CCT​GAT​GAG​ATT-3′	62	47.6	92
			5′-CGC​TGA​GCA​AGA​TCC​CTA​AA-3′	62	50	
39	Janus kinase and microtubule interacting protein 2 (JAKMIP2)	CR390426	5′-GAC​TGC​ATC​AGT​TCA​TCA​TTT​CTC-3’	62	41.7	130
			5’-ACA​GGA​ACA​CAT​TGC​TGG​T-3′	62	47.4	
40	Janus kinase and microtubule interacting protein 3 (JAKMIP3)	XM_426548	5′-TAT​CAA​CTT​CCA​CCA​CGT​TCC-3′	62	47.6	100
			5′-CAT​CAG​CTC​TGC​CAC​TAC​TAT​G-3′	62	50	
41	Leiomodin 3 (fetal) (LMOD3)	BX935813	5′-GAG​AAT​GAC​TGC​AGA​GGA​GAT​G-3′	62	50	97
			5′-TTT​GTA​GTG​CCG​CTC​CTT​C-3′	62	52.6	
42	Musculoskeletal, embryonic nuclear protein 1 (MUSTN1)	NM_213580	5′-CCA​AGT​CAT​GAA​GCA​GTG​TGA-3′	63	47.6	94
			5′-TGA​CTT​CTC​AAA​GAC​CGT​TTC​G-3′	63	45.5	
43	Myosin binding protein C, fast type (MYBPC2)	NM_001044659	5′-CTG​ATG​GAG​CGC​AAG​AAG​AA-3′	62	50	105
			5′-GAA​GAC​GCC​CTC​GAT​CAT​TT-3′	62	50	
44	Myosin binding protein C, slow type (MYBPC1)	BX935207	5′-CCT​GAA​ACG​TAG​GGA​GGT​TAA​A-3′	62	45.5	131
			5′-TGC​CTC​TCA​GGT​CAG​TGA​TA-3′	62	50	
45	Perilipin 1 (PLIN1)	NM_001127439	5′-CCA​GAA​GAG​GAG​GAG​GAA​GAT-3′	62	52.4	100
			5′-TAG​CAC​TGT​GAG​CCC​TGT​A-3′	62	52.6	
46	Phospholamban (PLN)	NM_205410	5′-CGA​TAG​CAG​GGT​TTC​CAT​ACT​T-3′	62	45.5	117
			5′-TGT​CAG​CTC​TCT​CCA​GTA​GAA-3′	62	47.6	
47	RCD1 required for cell differentiation1 homolog (S. pombe) (R*S. pombe*	001006521	5′-TGA​TTG​GAG​CCT​TGG​TGA​AA-3′	62	45	105
			5′-GTT​CAC​TGC​CAG​ACT​CCA​TAA​T-3′	62	45.5	
48	Slow muscle troponin T (TNNT1)	NM_205114	5′-CCC​TCC​ACA​TTG​AGC​ACA​T-3′	62	52.6	104
			5′-CTC​CAT​CAG​GTC​GAA​CTT​CTC-3′	62	52.4	
49	Troponin T type 3 (skeletal, fast) (TNNT3)	NM_204922	5′-GAA​GCA​AAC​AGC​TAG​AGA​GAC​A-3′	62	45.5	125
			5′-GGT​ATA​ACC​AGT​CCC​ACA​GTT​C-3′	62	50	
50	Troponin I type 1 (skeletal, slow) (TNNI1)	BX931462	5′-TCT​CTT​CGT​CCA​CAA​TCT​CAA​C-3′	62	45.5	128
			5′-ACA​GTC​GGA​GAA​GGA​GAG​ATA​C-3′	62	50	
51	Myostatin (MSTN)	NM_001001461.1	5′-GGA​TGG​GAC​TGG​ATT​ATA​GCA​C-3′	62	50	97
			5′-GGT​GAG​TGT​GCG​GGT​ATT​T-3′	62	52.6	
52	Follistatin (FST)	NM_205200.1	5′-ACA​ACT​TAT​CCG​AGC​GAG​TG-3′	62	50	112
			5′-CTT​CCT​CTG​GGT​CTT​CGT​TAA​T-3′	62	45.5	
53	Activin A receptor type 2A (ACVR2A)	NM_205367.1	5′-GCA​AGA​ATG​TGC​TGC​TGA​AA-3′	62	45	109
			5′-CCA​ACC​TGT​CCA​TGT​GTA​TCT-3′	62	47.6	
54	Activin A receptor type 2B (ACVR2B)	NM_204317.1	5′-GAA​GTG​TTA​GAG​GGA​GCA​ATC​A-3′	62	47.5	118
			5′-CTG​GAC​CAT​CAA​CTG​CTC​TAC-3′	62	52.4	
55	SMAD family member 2-Z (SMAD2Z)	NM_204561.1	5′-GGG​AGT​GCG​TCT​CTA​TTA​CAT​C-3′	62	50	110
			5′-CAG​GAT​GCC​AGC​CAT​ATC​TT-3′	62	50	
56	Activin A receptor type 1B (ACVR1B)	XM_015300267.2	5′-GCA​CGG​ATC​TCT​CTT​TGA​CTA​C-3′	62	50	120
			5′-TGA​GTA​CCC​ACG​ATC​TCC​AT-3′	62	50	
57	Transforming growth factor beta receptor 1 (TGFBR1)	NM_204246.1	5′-TCG​TGT​GCC​AAG​TGA​AGA​AG-3′	62	50	102
			5′-CCA​GAG​CCT​GAA​GTT​GTC​ATA​TC-3′	63	47.8	
58	Myogenin (MYOG)	NM_204184.1	5’-GGC​TGA​AGA​AGG​TGA​ACG​AA-3’	62	50	116
			5’-GCG​CTC​GAT​GTA​CTG​GAT​G-3’	62	57.9	
59	Mitogen-activated protein kinase kinase 6 (MAP2K6)	XM_003642348.2	5′-CTC​AGC​AGA​G′TCG​TCG​ATT​T-3′	62	47.6	101
			5′-GCA​GGG​TGA​AGA​AAG​GAT​GT-3′	62	50	
60	Mitogen-activated protein kinase kinase kinase 7 (MAP3K7)	XM_015284683.2	5′-CAG​CCC​TTG​TTT​CAG​GAG​AAG-3′	63	52.4	101
			5′-GCC​TCG​TTT​AGG​CTT​GGA​ATA​G-3′	63	50	
61	Caveolin 3 (CAV3)	NM_204370.2	5′-GCT​TTG​ATG​GTG​TCT​GGA​AAG-3′	61	47.6	142
			5′-ATG​TGG​CAG​AAG​GAG​ATG​AG-3′	61	50	
62	Protein kinase AMP-activated catalytic subunit alpha 1 (PRKAA1)	NM_001039603.1	5′-CTT​GAC​GAT​CAC​CAT​CTG​TCT​C-3′	62	50	140
			5′-TGC​CAC​TTC​GCT​CTT​CTT​AC-3′	62	50	
63	Protein kinase AMP-activated catalytic subunit alpha 2 (PRKAA2)	NM_001039605.1	5′-GGA​GGT​CTG​TGA​GAA​GTT​TGA​G-3′	62	50	124
			5′-GTT​CAT​GAT​CCT​CCG​GTT​GT-3′	62	50	
64	Creatine kinase, M-type (CKM)	NM_205507.1	5′-CGA​CCA​CTT​CCT​GTT​CGA​TAA-3′	62	47.6	109
			5′-GAA​CGT​CTT​GTT​GTC​GTT​GTG-3′	62	47.6	
65	Mechanistic target of rapamycin (serine/threonine kinase) (MTOR)	XM_417614.5	5′-AAG​GTT​TCT​TCC​GGT​CCA​TAT​C-3′	62	45.5	98
			5′-ATC​AGG​CCA​GTG​ACC​ATA​ATC-3′	62	47.6	
66	Ribosomal protein S6 kinase A1 (RPS6KA1)	NM_001109771.2	5′-GGA​ACC​CAG​CCA​ACA​GAT​TA-3′	62	50	104
			5′-TTC​CCT​TCG​GTA​CAG​CTT​ATT​C-3′	62	45.5	
67	Carnitine palmitoyltransferase I (CPT1)	DQ314726.1	5′-GCC​TTC​GTG​CGC​AGT​AT-3′	62	58.8	146
			5′-ACG​TAG​AGG​CAG​AAG​AGG​T-3′	62	52.6	
68	Acyl-CoA synthetase long-chain family member 1 (ACSL1)	NM_001012578.1	5′-GCC​AGT​ACG​TAG​GCA​TCT​TT-3′	62	50	116
			5′-TGC​TTC​AGT​TCC​CAG​TGT​ATC-3′	62	47.6	
69	Enoyl-CoA hydratase, short chain 1 (ECHS1)	NM_001277395.1	5′-CAG​GTG​GGA​GCT​ATT​GTC​ATC-3′	62	52.4	97
			5′-CAT​AGC​ACT​CCT​GGA​AGG​TTT-3′	62	47.6	
70	Hydroxyacyl-CoA dehydrogenase (HADH)	NM_001277897.1	5′-GCT​ATC​CCA​TGG​GTC​CAT​TT-3′	62	50	100
			5′-AGA​GGA​TTG​TTG​GGC​TCT​ATT​G-3′	62	45.5	
71	Acyl-CoA oxidase 2 (ACOX2)	XM_015293306.2	5′-TGC​CAC​CAT​CTG​TCA​CTT​ATC-3′	62	47.6	141
			5′-TAG​CTG​CTG​TGC​TGC​TTA​TC-3′	62	50	
72	Sterol regulatory element binding protein 1 (SREBP1)	AJ310768.1	5′-CAT​GGA​GGT​GGC​GAA​GG-3′	62	64.7	134
			5′-TGT​CAG​GCT​CGG​AGT​CA-3′	62	58.8	
73	Fibroblast growth factor 2 (FGF2)	NM_205433.1	5′-TTC​GAG​CGC​TTG​GAA​TCT​AAT​A-3′	62	40.9	94
			5′-GCT​TGT​ACT​GTC​CAG​TCC​TTT-3′	62	47.6	
74	Fibroblast growth factor receptor 1 (FGFR1)	NM_205510.1	5′-CGT​CAC​CAA​AGT​GGC​TGT​A-3′	62	52.6	98
			5′-TGC​CGA​TCA​TCT​TCA​TCA​TCT​C-3′	62	45.5	
75	DNA methyltransferase 3 alpha (DNMT3A)	NM_001024832.1	5′-CCT​TCT​TCT​GGC​TCT​TTG​AGA​A-3′	62	45.5	111
			5′-CAG​ACA​CCT​CTT​TGG​CAT​CA-3′	62	50	
76	Forkhead box O3 (FOXO3)	MK861853.1	5′-CTC​TCA​GGC​TCC​TCT​TTG​TAT​TC-3′	62	47.8	109
			5′-CAC​ACT​CCA​AGC​TCC​CAT​T-3′	62	52.6	
77	Peroxisome proliferator–activated receptor gamma (PPARγ)	AF163811.1	5′-CCC​AAG​TTT​GAG​TTT​GCT​GTG-3′	62	47.6	99
			5′-TGG​GCG​ATC​TCC​ACT​TAG​TA-3′	62	50	
78	Myogenic factor 6 (MYF6)	FJ882409.1	5′-GCT​GGA​TCA​GCA​GGA​CAA​A-3′	62	52.6	100
			5′-GCA​GGT​GCT​CAG​GAA​GTC-3′	62	61.1	
79	Acetyl-CoA carboxylase α(ACACA)	NM_205505.1	5′-CAG​ATT​TGT​TGT​CAT​GGT​GAC-3′	60	42.9	162
			5′-ACA​GCC​TGC​ACT​GGA​ATG​C-3′	60	57.9	
80	Acetyl-CoA carboxylase β(ACACB)	XM_025155692.1	5′-GCT​CCT​GCT​GCC​CAT​ATA​TTA-3′	60	47.6	94
			5′-GTC​CGT​GAT​GAC​ACC​TTT​CT-3′	60	50	
81	Fatty acid synthase (FASN)	NM_205155.3	5′-GTT​CTC​TGT​ACA​GAG​AAT​GTG-3′	60	42.9	168
			5′-CCA​TGT​TTG​ACT​TGG​TTG​ATC-3′	60	42.9	

### The statistical analysis

To assess the expression variation of the candidate reference genes, all the samples were divided into three broad categories: the combination of 7EB and 18TM samples of control broiler and improved Aseel, 7EB and 18TM samples of control broiler alone, and 7EB and 18TM samples of improved Aseel alone. The qRT-PCR machine–generated Ct values for each of the cDNA samples were then used to determine the degree of data variability between the samples. The stability level of the 24 candidate reference genes from the 7EB and 18TM of control broiler and improved Aseel was determined using five statistical algorithms: geNorm, NormFinder, BestKeeper, Delta CT, and RefFinder (Vandesompele et al., 2002; Andersen et al., 2004; Pfaffl et al., 2004; Silver et al., 2006; Xie et al., 2012). GeNorm and NormFinder, which use relative expression values as input data and convert Ct values to linear scale expression quantities using the 2^−∆Ct^ method, as well as BestKeeper, which uses the raw Ct value directly and the comparative Ct method, were used to determine the expression stability level. RefFinder, a program that combines the most important computational tools currently available (geNorm, Normfinder, BestKeeper, and the comparative 2^−∆Ct^ method) to compare and rank the stability of candidate reference genes with the geometric mean of individual gene appropriate weight, was used to determine the overall final ranking of reference genes across all samples. Comparison of mean expression values for qRT-PCR between the control broiler and PD4 improved Aseel groups were made using the Student’s t-test and *p* ≤ 0.05 was considered statistically significant.

## Results

### Source, selection, primer design, and verification of candidate reference genes

In the present study, to identify the suitable reference genes for the 7th- and 18th-day embryos of the control broiler and improved Aseel, 24 candidate reference genes with a wide range of biological functions were selected based on previous studies of various avian and non-avian species. These are 18S rRNA, ALB, B2MG, β-ACT, EEF1A1, GAPDH, GUSB, HMBS, HSP10, HSP70, L-LDBC, MRPS27, MRPS30, PGK2, PPP2CB, RPL5, RPL13, RPL14, RPL19, RPL23, SDHA, TBP, TFRC, and DNAJC24. The chicken orthologous genes were obtained from NCBI, and the CDS region was found and amplified with gene-specific primers (Supplementary Table S1). For all the primer pairs, the melting curve analysis was performed to confirm the specific amplification for each reference gene, a single peak with no visible primer-dimer formation and genomic DNA contamination was observed, and no signals were detected in the non-template controls.

### Expression stability and ranking of candidate reference genes

**FIGURE 2:**
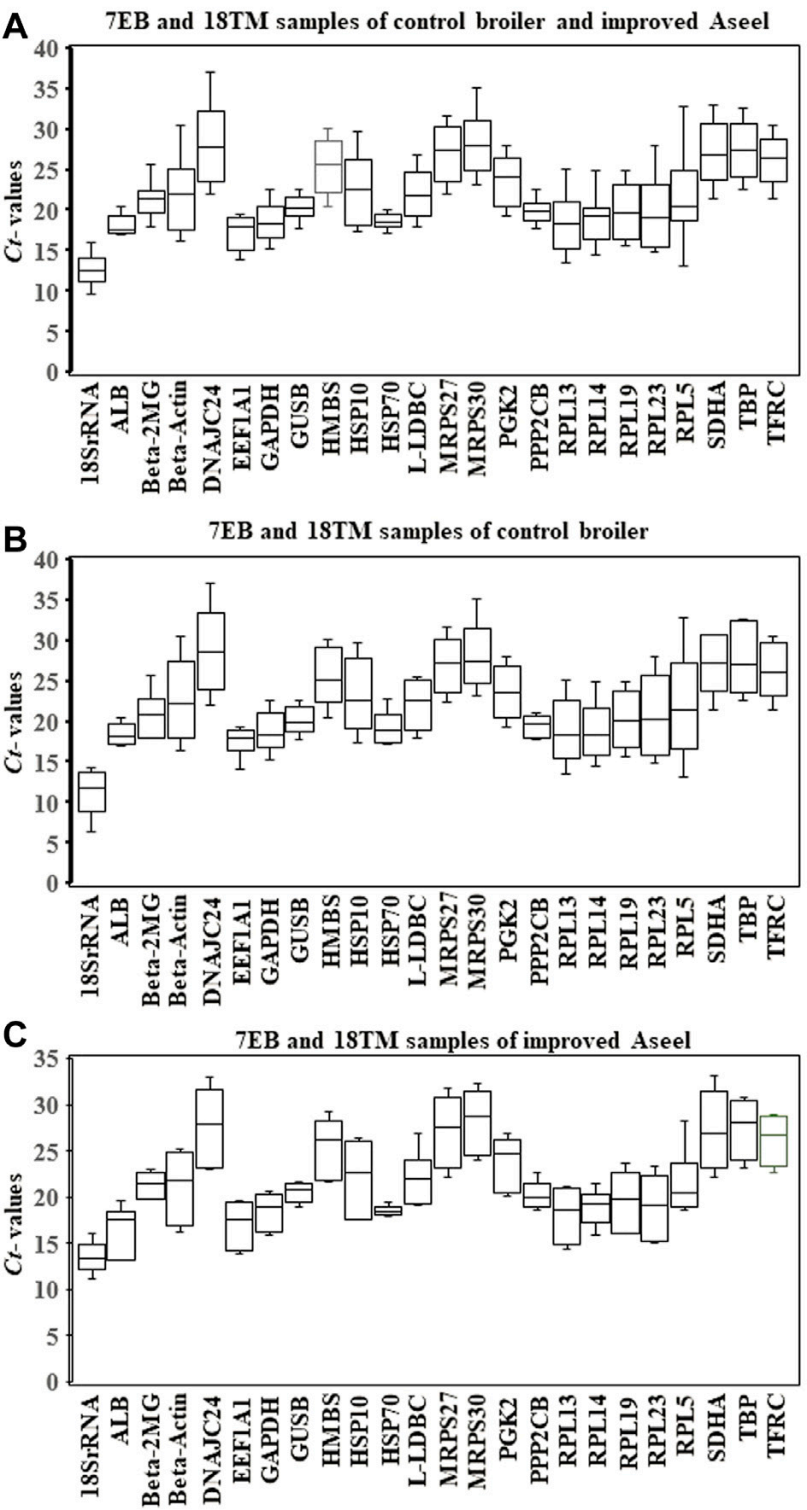
Relative level of mRNA expression of the 24 candidate reference genes in the 7EB and 18TM of both control broiler and improved Aseel. The data are presented as mean cycle threshold (Ct) values and shown as box and whisker plots. The boxes represent the interquartile range of the mean Ct values, whereas the middle, up, and lower bars represent the mid-hinge, maximum, and minimum Ct values, respectively. The *X*-axis represents the gene names, and *Y*-axis represents the Ct values of all the tissues. **(A)** 7EB and 18TM samples of control broiler and improved Aseel; **(B)** 7EB and 18TM samples of control broiler; **(C)** 7EB and 18TM samples of PD4.

**FIGURE 3:**
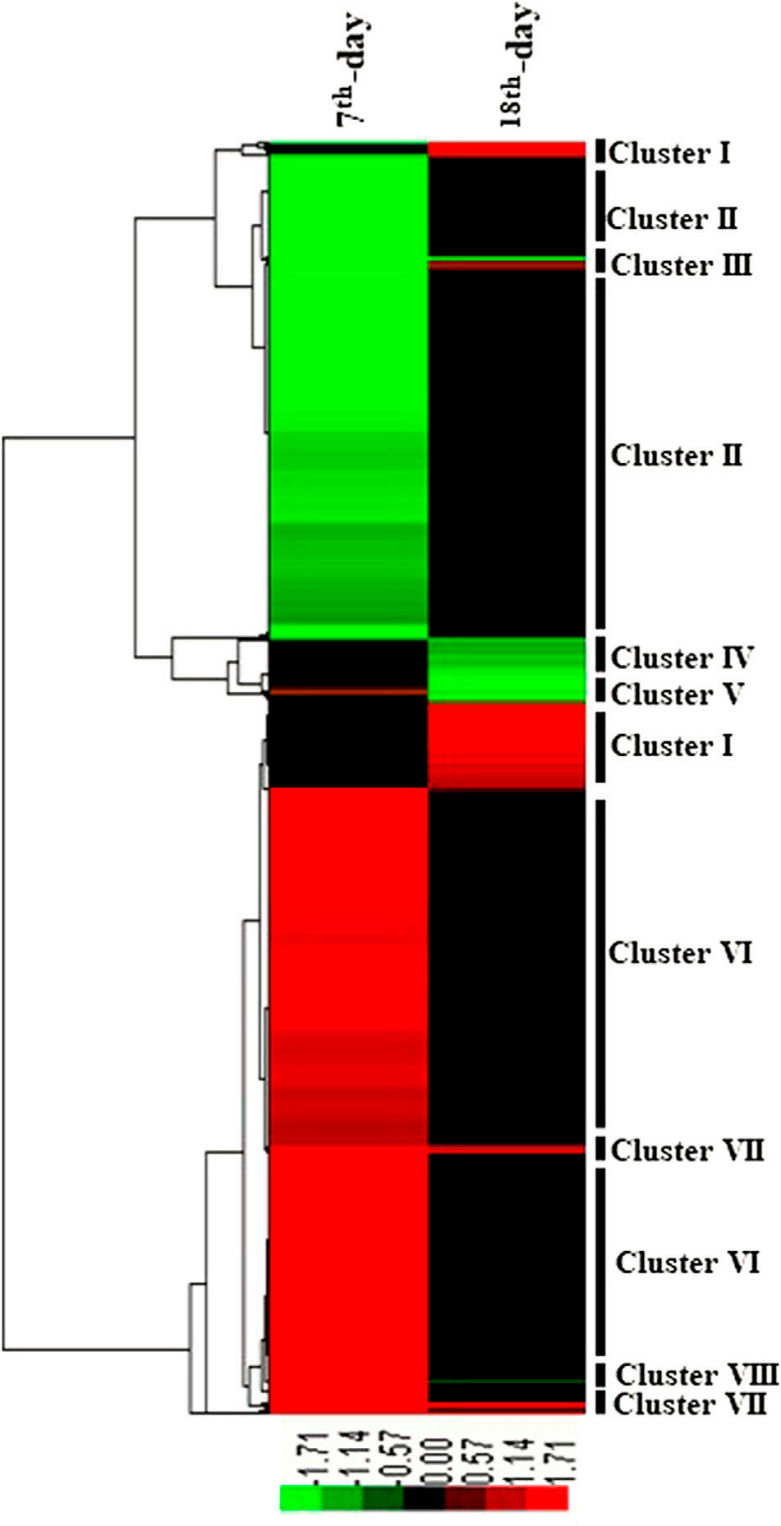
Hierarchical clustering of DEGs on the 7EB and 18TM of the embryo (Aseel vs. control broiler). Hierarchical clustering of differentially expressed genes led to the formation of eight distinct clusters: I, II, III, IV, V, VI, VII, and VIII, which include genes up- and downregulated, defining the specific molecular regulation of Aseel growth. Each row represents the expression pattern of a single gene, and each column corresponds to a single sample: column 1, 7EB; column 2, 18TM. The expression levels are represented by a color chat (*p* value ≤0.05 and fold change ≥1), with red representing upregulation, green representing downregulation, and black representing the missing values or no change.

Expression levels of all candidate reference genes were measured in the samples collected from the 7EB and 18TM of the control broiler and improved Aseel. Each reference gene had different expression ranges across all sample sets, and the 18S rRNA and DNAJC24 genes showed the most (Ct = 12.34) and the least (Ct = 33.88) abundant transcripts, respectively. In the combined analysis, we observed that not all selected reference genes were expressed uniformly across 7EB and 18TM of the control broiler and improved Aseel. The genes with the lowest global variability were GUSB, PP2CB, and HSP70 (Figure 2A). The results show that the GUSB reference gene had the least variation in expression, with mean Ct values ranging from 17.78 to 22.52, whereas the RPL5 gene showed a much higher expression variation, with mean Ct values ranging from 13.07 to 32.89 across all sample sets (Figure 2A). In control broiler samples, PP2CB, ALB, and GUSB were the top three genes with the lowest variation (Figure 3B); whereas HSP70, GUSB, and β-2MG showed little variation in improved Aseel (Figure 3C). It is important to note that there was a wide range of variation among selected reference genes, and it shows that not a single reference gene was expressed constantly across the 7EB and 18TM of control broiler and improved Aseel in the present study. Therefore, it is essential to choose the most reliable reference gene for expression profiling gene/s in different embryos of the control broiler and improved Aseel. The most popular statistical tools geNorm, NormFinder, BestKeeper, Delta CT, and RefFinder were used for the analysis to choose the best and most trustworthy reference gene and rank all the potential reference genes according to their stability values for accurate gene expression (Table 2). The variation among the reference genes determined by geNorm is stability measure (M value) and pairwise comparison expression ratio and provides an optimal number of genes in a given experiment. NormFinder measures the reference gene stability by overall expression variation and across samples variation to reduce sensitivity toward co-regulation. BestKeeper calculates the gene expression variation based on Ct values, calculates the Pearson correlation coefficient by pairwise correlation analysis for all reference genes, and finds the stable genes. The Delta CT method directly used the raw Ct values and found the best stable genes. RefFinder is conclusive of the calculations using the aforementioned algorithms and suggested stable genes.

**TABLE 2:** Ranking of the candidate reference genes according to their stability value per indicated software

Gene Name	geNorm						NormFinder						BestKeeper								ΔCT							Comprctensive						
	7EB & 18TM of CB & PD4 combined analysis		7EB & 18TM of CB analysis		7EB & 18TM of PD4 analysis		7EB & 18TM of CB & PD4 combined analysis		7EB & 18TM of CB analysis		7EB & 18TM of PD4 analysis		7EB & 18TM of CB & PD4 combined analysis			7EB & 18TM of CB analysis			7EB & 18TM of PD4 analysis		7EB & 18TM of CB & PD4 combined analysis		7EB & 18TM of CB analysis		7EB & 18TM of PD4 analysis			7EB & 18TM of CB & PD4 combined analysis			7EB & 18TM of CB analysis		7EB & 18TM of PD4 analysis	
	M	R	M	R	M	R	SV	R	SV	R	SV	R	SD	R	SD		R	SD		R	SD	R	SD	R		SD	R		GM	R	GM	R	GM	R
18S rRNA	2.05	18	2.27	18	1.70	18	3.02	19	3.25	18	2.26	19	1.83	6	2.13		7	1.35		5	3.75	20	4.09	19		2.96	21		14.43	18	14.6	18	13.95	18
ALB	3.00	23	2.48	19	2.60	23	5.99	24	3.22	17	7.95	24	2.54	10	1.13		**2**	4.56		24	6.32	24	3.86	18		8.08	24		19.28	23	10.52	12	24	24
B2MG	2.70	22	3.27	23	1.82	19	4.02	23	5.48	24	2.27	20	1.66	4	2.25		8	0.99		**3**	4.4	23	5.63	24		2.93	20		14.85	19	18.24	22	12.45	16
βActin	1.48	13	1.64	14	1.04	11	2.38	16	2.74	16	2.01	16	4.12	23	4.52		22	3.72		22	2.88	16	3.18	16		2.44	14		16.94	21	17.05	21	15.59	20
DNAJC24	1.60	15	1.72	15	1.16	13	2.71	17	3.25	19	2.28	21	4.37	24	4.98		23	3.77		23	3.13	17	3.59	17		2.63	17		18.25	22	18.57	23	18.41	22
EEF1A1	1.54	14	1.83	16	0.48	**2**	0.87	4	1.25	6	0.33	**2**	1.73	5	1.39		4	2.08		8	2.43	9	2.85	13		1.86	**3**		7.21	6	8.53	7	3.46	**3**
GAPDH	1.18	8	1.23	8	1.10	12	0.37	**1**	0.06	**1**	0.57	4	1.91	7	1.97		6	1.85		7	2.28	5	2.52	4		2.01	7		4.21	**3**	3.83	**3**	7.1	6
GUSB	2.38	20	2.86	21	1.99	21	3.07	20	3.83	21	2.40	22	1.14	**3**	1.29		**3**	0.89		**2**	3.68	19	4.28	21		3.02	22		12.44	15	13.06	16	12.08	15
HMBS	0.67	**2**	0.54	**2**	0.91	8	1.02	5	0.87	4	1.20	9	3.01	14	3.21		14	2.82		16	2.24	**3**	2.37	**3**		2.08	10		5.01	4	4.74	4	10.67	12
HSP10	1.01	6	0.97	5	0.99	10	1.86	14	2.0	14	1.81	14	3.79	21	4		20	3.58		21	2.54	12	2.73	10		2.29	13		12.54	16	11.38	13	14.32	19
HSP70	2.54	21	3.06	22	1.91	20	3.55	21	4.76	23	2.23	18	1.08	**1**	1.62		5	0.42		**1**	4.04	22	5.01	23		2.92	19		10.04	10	15.71	19	9.21	8
L-LDBC	1.32	10	1.42	10	1.28	15	1.09	6	1.31	7	0.95	7	2.45	9	2.74		9	2.16		9	2.45	10	2.72	8		2.19	12		8.78	8	8.63	8	10.49	11
MRPS27	1.10	7	1.32	9	0.95	9	1.36	10	1.21	5	1.57	13	3.08	16	2.94		13	3.22		19	2.43	8	2.62	6		2.18	11		10.06	11	7.9	6	12.84	17
MRPS30	0.92	5	1.03	6	0.77	5	1.48	12	1.80	13	1.20	8	3.26	18	3.43		17	3.10		18	2.42	7	2.74	11		1.96	6		9.76	9	11.42	14	8.49	7
PGK2	0.38	**1**	0.35	**1**	0.41	**1**	0.52	**3**	0.51	**3**	0.48	**3**	2.59	11	2.8		10	2.38		11	2.13	**2**	2.35	**2**		1.82	**2**		2.85	**2**	2.78	**2**	2.85	**2**
PPP2CB	2.23	19	2.69	20	1.56	17	2.91	18	3.80	20	1.86	15	1.11	**2**	1.12		**1**	1.1		4	3.55	18	4.25	20		2.67	18		10.67	13	9.57	11	11.81	14
RPL13	0.83	4	0.75	**3**	0.82	6	1.15	7	1.47	9	0.82	5	3.06	15	3.39		16	2.72		14	2.26	4	2.54	5		1.89	4		6.77	5	7.33	5	6.65	5
RPL14	1.66	16	1.13	7	1.41	16	1.45	11	1.46	8	1.54	12	2.15	8	2.86		11	1.42		6	2.66	13	2.72	9		2.55	15		11.81	14	8.92	9	11.64	13
RPL19	1.25	9	1.53	12	0.62	**3**	1.33	8	1.76	12	0.87	6	2.92	13	3.24		15	2.60		13	2.46	11	2.9	14		1.91	5		10.34	12	13.45	17	6.28	4
RPL23	1.43	12	1.59	13	0.70	4	2.09	15	2.60	15	1.22	11	3.64	20	4.46		21	2.82		15	2.77	15	3.09	15		2.04	9		15.55	20	16.04	20	9.28	9
RPL5	1.83	17	2.03	17	2.10	22	3.59	22	4.52	22	2.83	23	3.93	22	5.32		24	2.54		12	4.04	21	4.75	22		3.43	23		20.68	24	21.38	24	19.55	23
SDHA	1.37	11	1.48	11	1.22	14	1.84	13	1.68	11	2.11	17	3.45	19	3.43		18	3.47		20	2.7	14	2.82	12		2.60	16		14.27	17	12.99	15	16.90	21
TBP	0.78	**3**	0.88	4	0.87	7	1.35	9	1.61	10	1.21	10	3.23	17	3.57		19	2.89		17	2.35	6	2.66	7		2.03	8		7.78	7	9.03	10	10.21	10
TFRC	0.38	**1**	0.35	**1**	0.41	**1**	0.4	**2**	0.49	**2**	0.27	**1**	2.59	12	2.88		12	2.31		10	2.1	**1**	2.33	**1**		1.79	**1**		2.21	**1**	2.21	**1**	1.78	**1**

M*,* gene expression stability measure; SD*,* standard deviation value; SV, stability value; GM*,* geomean value; and R, ranking

### Differentially expressed transcripts during embryo development stages

To study the effect of muscle development, genome-wide expression analysis was carried out at muscle initiation (7th-day embryo) and muscle development (18th-day thigh muscle) stages (Figures 1A,B). Labeled RNA was hybridized to the Affymetrix GeneChip™ Chicken Genome Array. After statistical data analysis, transcripts with an FDR-adjusted *p*-value ≤ 0.05 and a fold change ≥ 1 were considered as differentially expressed transcripts (DETs) (Figure 1C). The complete list of the DETs in improved Aseel during embryo development stages as compared to their respective control broiler samples is presented in Supplementary Material S1. In total, 8,069 transcripts, which accounted for approximately 24% of the total transcripts present on the GeneChip™ Chicken Genome Array, showed differential expression in improved Aseel at various stages analyzed. The maximum number of transcripts (6,896, 21% of total DETs) showed differential expression on the 7th day of the embryo, and the least number of transcripts (1,173, 3.5% of total DETs) showed differential expression on the 18th day of the embryo. Commonly up- and downregulated muscle-responsive transcripts were identied among the embryo development stages to nd out the degree of overlap (Figures 1D,E). The maximum unique number of upregulated (3,799) and downregulated transcripts (2962) was observed in a 7th-day embryo. A small number of upregulated (654) and downregulated (384) transcripts were uniquely differentially expressed on the 18th-day of the thigh muscle. The commonly differentially expressed (91 upregulated and 44 downregulated) transcripts were identified among the embryo development stages, respectively (Figures 1D,E; Supplementary Material S1).

### Cluster analysis of differentially expressed transcripts

To profile the gene expression patterns in response to muscle slow growth and egg production during embryo development, the 8,069 DETs were classied using hierarchical clustering. The expression patterns were separated into eight major clusters (I–VIII) based on tree branching (Figure 3). Transcripts and involved pathways present in each stage within each cluster are presented in Supplementary Material S2. Among the eight major clusters, upregulated transcripts were enriched in clusters I, VI, and VII, and downregulated transcripts were enriched in clusters II, III, and IV.

### GO annotation and pathway enrichment analysis of differentially expressed genes

DAVID 6.7 was used to annotate and enrich the DEGs related to GO annotations and pathways between the 7th-day up and the 18th-day down and *vice versa*. The results showed that coenzyme metabolism, cell division and chromosome partitioning, outer membrane, and transcription/cell division and chromosome partitioning functions were upregulated in the 7th-day embryo and downregulated in the 18th-day thigh muscle, whereas chaperones, lipid metabolism, outer membrane/carbohydrate transport and metabolism, protein turnover, and posttranslational modification functions were downregulated in the 7th-day embryo and upregulated in the 18th-day thigh muscle. Cell envelope biogenesis, cytoskeleton, and inorganic ion transport and metabolism functions were differentially regulated between the 7th-day embryo and 18th-day thigh muscle compared to the respective controls (Supplementary Material S3). The KEGG pathway enrichment analysis of DEGs was performed using the IPA tool. The results showed that differential regulation of pathways between 7th-day embryo and 18th-day thigh muscle of PD4 compared to their respective controls, that is, Cell cycle, Cell adhesion molecules (CAMs), SNARE interactions in vesicular transport, Oocyte meiosis, Endocytosis, Apoptosis, ABC transporters, Calcium signaling pathway, MAPK signaling pathway, Wnt signaling pathway, Jak-STAT signaling pathway, Toll-like receptor signaling pathway, TGF-βsignaling pathway, cytokine–cytokine receptor interaction, Basal transcription factors, Focal adhesion, Tight junction, Regulation of actin cytoskeleton, Cardiac muscle contraction, Vascular smooth muscle contraction, Insulin signaling pathway, Oxidative phosphorylation, Glutathione metabolism, Glycolysis/Gluconeogenesis, Citrate cycle (TCA cycle), Pentose phosphate pathway, Pyruvate metabolism, Fatty acid biosynthesis, Fatty acid metabolism, Glycerophospholipid metabolism, Heparan sulfate biosynthesis, N-Glycan biosynthesis, Purine metabolism, Pyrimidine metabolism, Tryptophan metabolism, Serine and threonine metabolism, and Valine, leucine and isoleucine degradation (Supplementary Materials S2, S3).

### Validation of DEGs by qPCR

**FIGURE 4:**
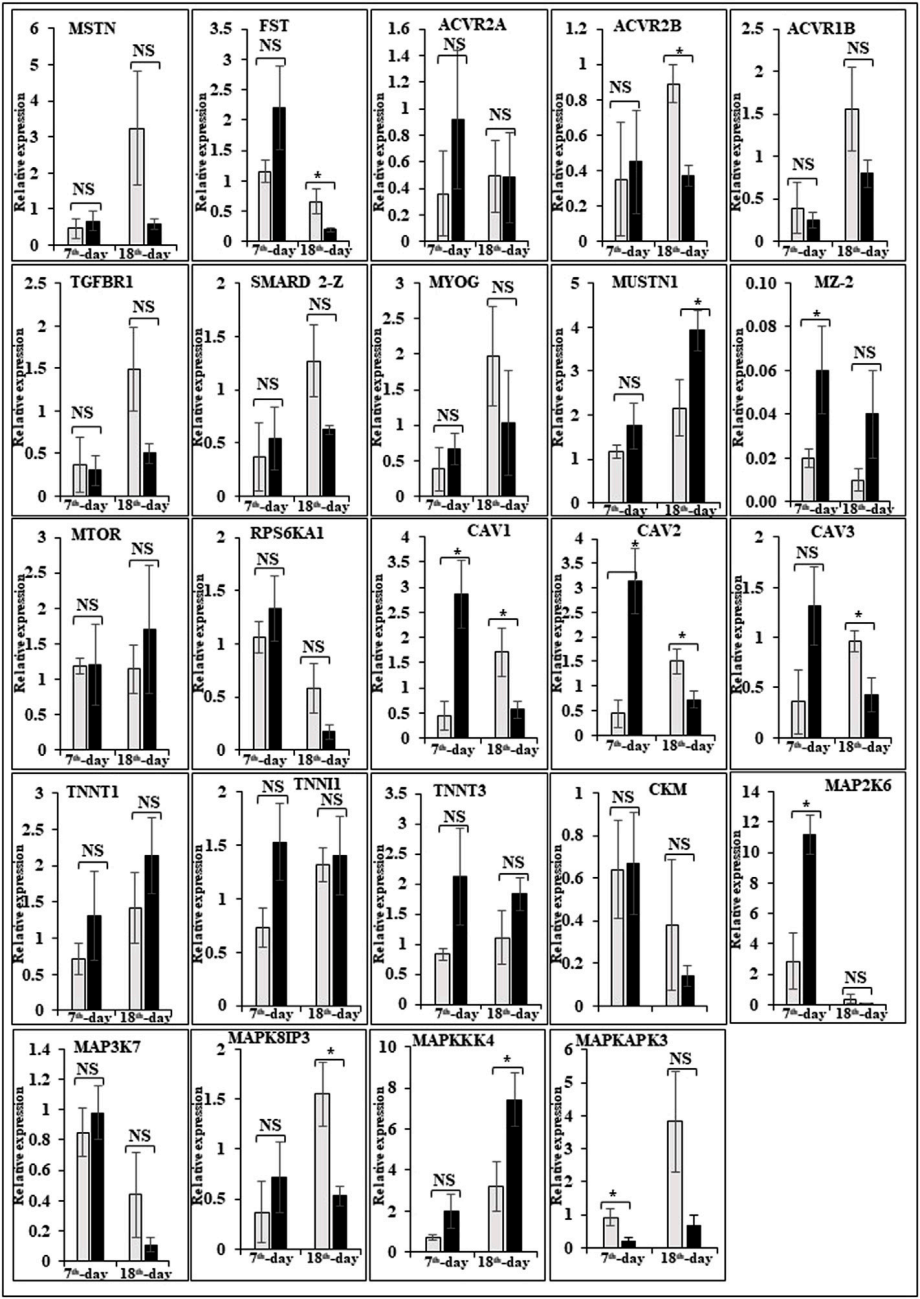
Relative expression of DEGs that are involved in muscle development, myostatin signaling, muscle metabolism (energy sensing and storage), and protein synthesis. The Y-axis represents relative mRNA expression level, and the X-axis represents tissue samples used for the qPCR study ( Control broiler, PD4). The *p* values have been stated on the comparative bars; * indicates the *p* ≤ 0.0 5, and NS indicates the non-significant. Standard error was used for error bars.

**FIGURE 5:**
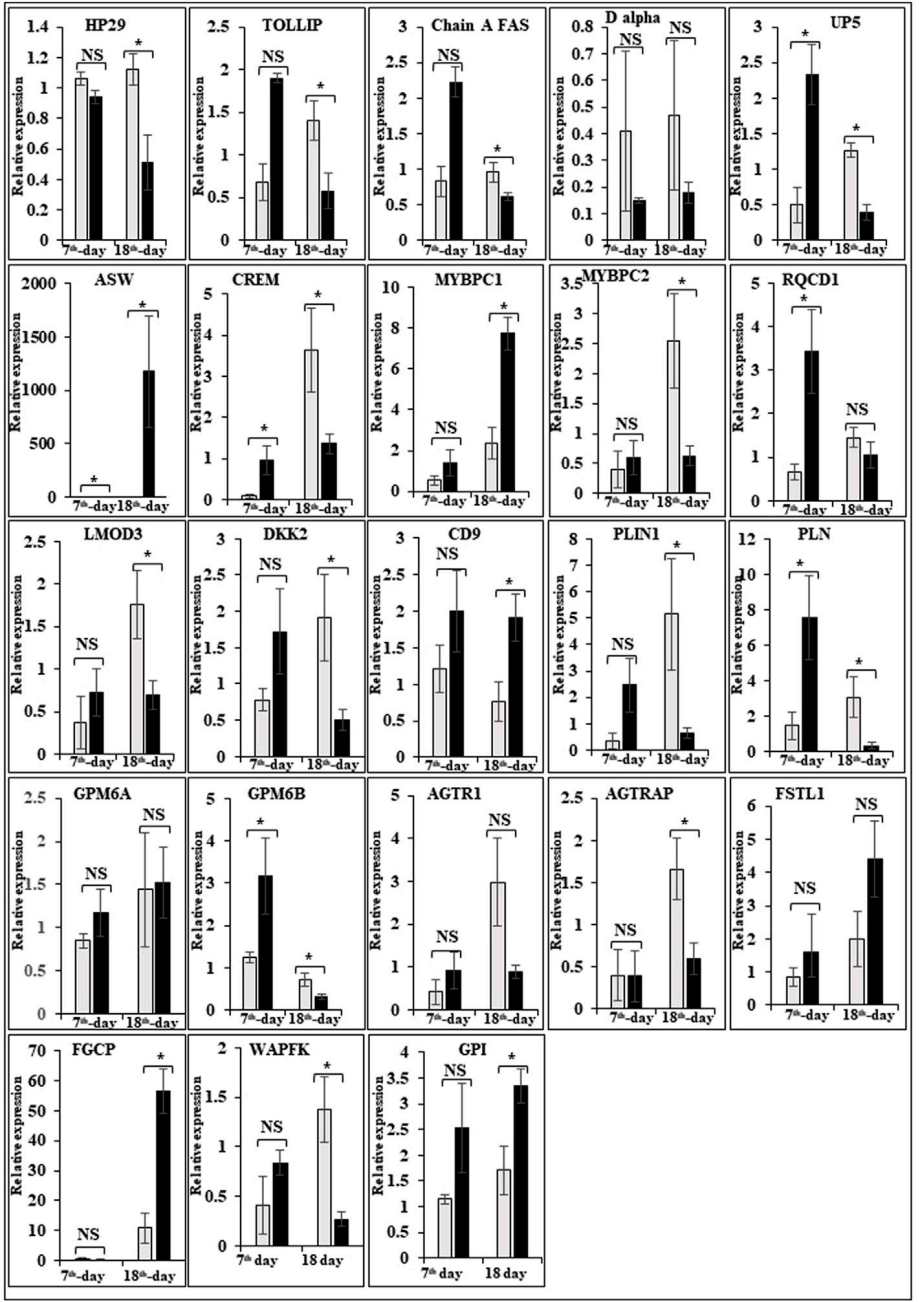
Relative expression of DEGs that are involved in embryo development. The Y-axis represents relative mRNA expression level, and the X-axis represents tissue samples used for the qPCR study ( Control broiler, PD4). The *p* values have been stated on the comparative bars; * indicates the *p* ≤ 0.05, and NS indicates the non-significant. Standard error was used for error bars.

**FIGURE 6:**
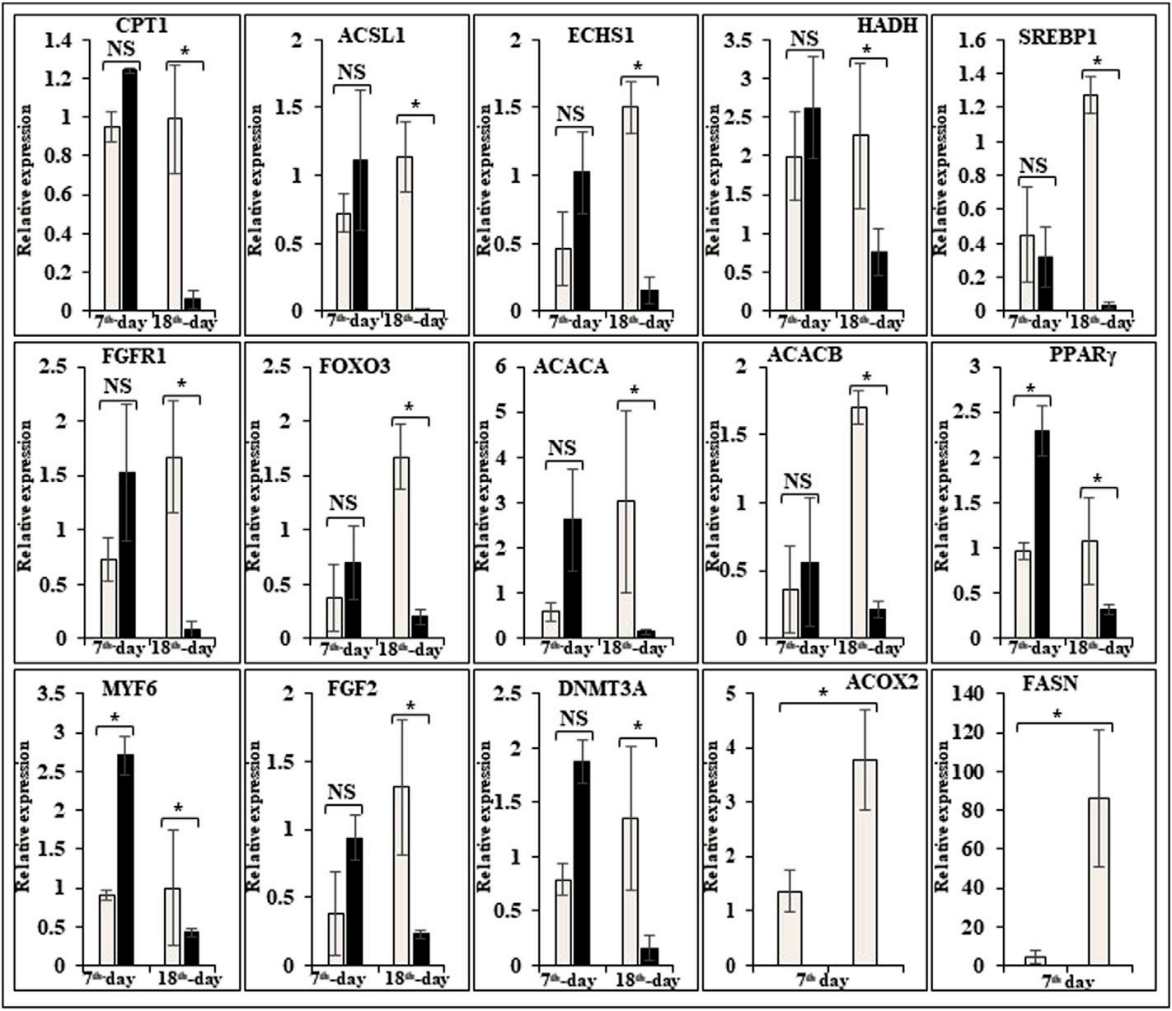
Relative expression of DEGs that are involved in fatty acid metabolism. The Y-axis represents relative mRNA expression level, and the X-axis represents tissue samples used for the qPCR study ( Control broiler, PD4). The *p* values have been stated on the comparative bars; * indicates the *p* ≤ 0.05, and NS indicates the non-significant. Standard error was used for error bars.

**FIGURE 7:**
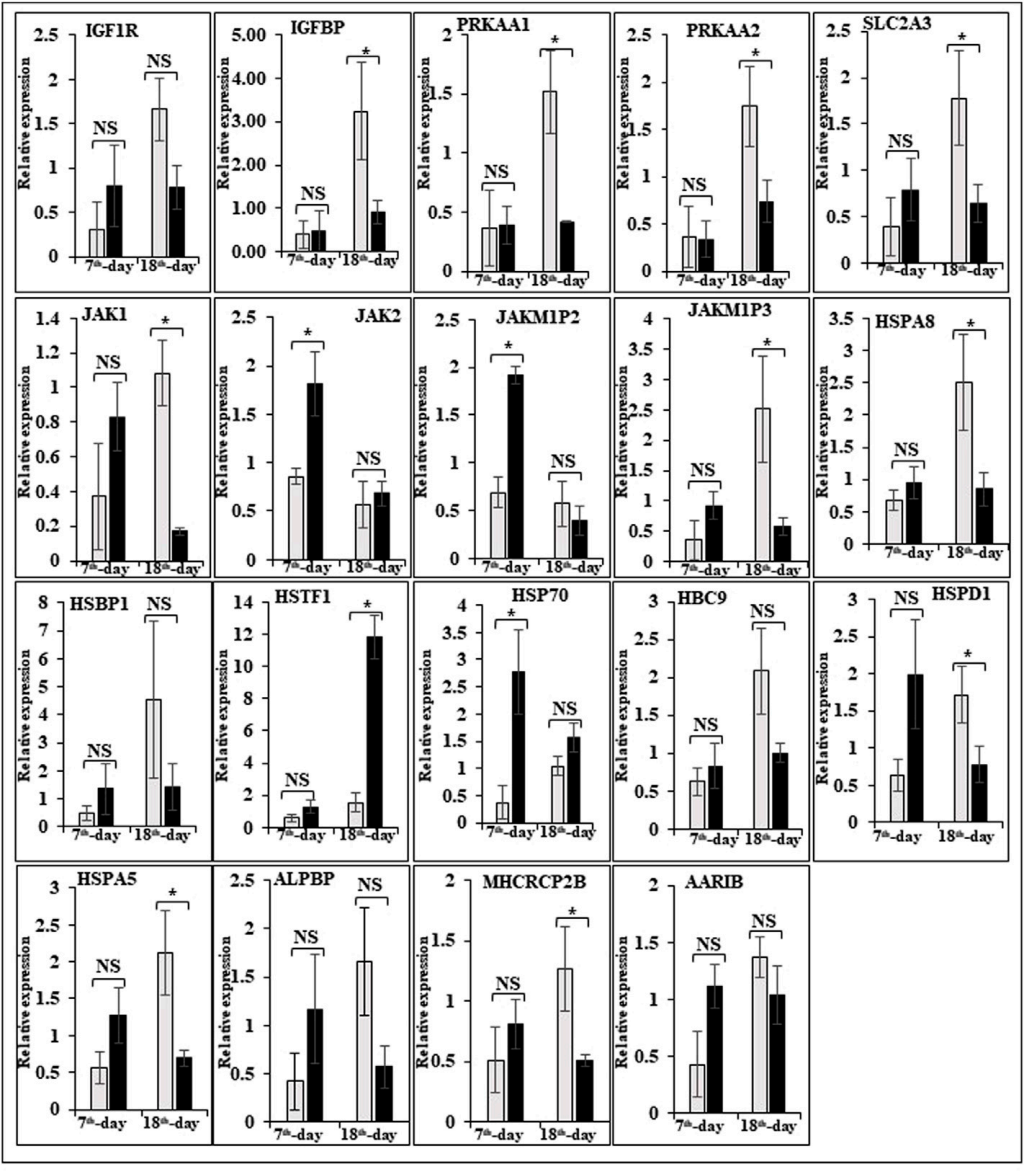
Relative expression of DEGs that are involved in cell signaling and egg production. The Y-axis represents relative mRNA expression level, and the X-axis represents tissue samples used for the qPCR study ( Control broiler, PD4). The *p* values have been stated on the comparative bars; * indicates the *p* ≤ 0.05, and NS indicates the non-significant. Standard error was used for error bars.

In this study, the expression of DEGs between the fast (CB) and slow growth (PD4) chickens of the 7th-day embryo and 18th-day thigh muscle was verified by qPCR (Supplementary Table S2). The verified transcripts were divided into four groups: i. muscle development, myostatin signaling, muscle metabolism, and protein synthesis (Figure 4), ii. Embryo development (Figure 5), iii. Fatty acid metabolism (Figure 6), and iv. Cell signaling and egg production (Figure 7). The results showed that the expression trend of the DEGs between the fast and slow-growing chickens is consistent in qPCR results, and this attests to the reliability of the microarray data.

## Discussion

Globally, chicken is one of the most protein-rich meat sources. Muscle development and egg production are essential genetic traits in commercially grown chickens. However, not much information is available on genes involved in muscle development and egg production in slow and fast-growing chickens. In this study, we selected fast (CB) and slow-growth (PD4) chickens to determine the expression of genes related to embryo initiation and developmental stages. Microarray was conducted with the 7th-day embryo (7EB) and 18th-day thigh muscle (18TM) of PD4 and CB, respectively. According to the MIQE guidelines, selecting suitable reference genes may vary for different species, varieties, experimental conditions, and tissues and has to be validated before gene expression study (Bustin et al., 2009). Previous and recent studies also described different expression patterns of reference genes and focused on validating reference genes applied to different avian tissues (Olias et al., 2014; Bages et al., 2015; Nascimento et al., 2015). However, so far, validation of genes for their stable expression patterns in different embryo tissues, such as 7th and 18th-day embryos of control broiler and improved Aseel has not been performed.

### Candidate reference genes validation

For accurate gene expression, to select the best and most reliable reference gene and rank all the candidate reference genes according to their stability value, the most commonly used statistical programs were used, that is, geNorm, NormFinder, BestKeeper, Delta CT, and RefFinder (De Boever et al., 2008; Yue et al., 2010; Yang F. et al., 2013; Olias et al., 2014; Bages et al., 2015; Nascimento et al., 2015; Borowska et al., 2016; Mitra et al., 2016; Khan et al., 2017; Zhang et al., 2018; Mogilicherla et al., 2022). These algorithms showed some differences in the stability ranking of stable reference genes, which may be due to the differences in each statistical program (Table 2). Ranking the stability of 24 genes is crucial, as is confirming the number of reference genes required for precise gene expression profiling in various embryonic tissues. For each gene, geNorm generates a stability measure (M value), allowing for ranking based on expression stability (with the lower value indicating increased gene stability across samples). To assess the value of including more references, it additionally provides a pairwise stability measure for the normalization (Exposito-Rodriguez et al., 2008; Paolacci et al., 2009). According to the geNorm stability criteria, the most stable genes in various analyses are: control broiler alone [TFRC (0.35), PGK2 (0.38), HMBS (0.54), and RPL13 (0.75)]; improved Aseel alone [TFRC (0.41), PGK2 (0.41), EEF1A1 (0.48), and RPL19 (0.62)]; and combined analysis [TFRC (0.38), PGK2 (0.38), HMBS (0.67), and TBP (0.78)], respectively were within the M value ≤ 1 threshold range, demonstrating a trustworthy stability (Table 2). To enable a direct estimate of expression variation, including ranking genes according to their stability using a model-based approach, NormFinder provides a stability measure and groups samples (Hellemans et al., 2007; Mitra et al., 2016). The results of an analysis using NormFinder revealed the relative rankings of the genes in various combinations, including control broiler alone [GAPDH (0.06), TFRC (0.49), and PGK2 (0.51)]; improved Aseel alone [TFRC (0.27), EEF1A1 (0.33), and PGK2 (0.48)]; and combined analysis [GAPDH (0.37), TFRC (0.4), and PGK2 (0.52)], respectively (Table 2). Based on Ct values, BestKeeper determines the variation in gene expression for each housekeeping gene. By using pairwise correlation analysis, BestKeeper calculates the Pearson correlation coefficient, estimates the inter-gene relationships, and identifies the stable genes in all combinations: control broiler alone [PPP2CB (1.12), ALB (1.13), GUSB (1.29)]; improved Aseel alone [HSP70 (0.42), GUSB (0.89), B2MG (0.99)]; combined analysis [HSP70 (1.08), PPP2CB (1.11), GUSB (1.14)], respectively (Table 2). The Delta CT results supported the findings of geNorm and NormFinder, and they revealed the best stable genes in all combinations: control broiler alone [TFRC (2.33), PGK2 (2.35), HMBS (2.37)]; improved Aseel alone [TFRC (1.79), PGK2 (1.82), EEF1A1 (1.86)]; combined analysis [TFRC (2.1), PGK2 (2.13), HMBS (2.24)], respectively (Table 2). RefFinder integrated the results from each of the aforementioned algorithms and suggested stable genes for all combinations: control broiler alone [TFRC (2.21), PGK2 (2.78), GAPDH (3.83)]; improved Aseel alone [TFRC (1.78), PGK2 (2.85), EEF1A1 (3.46)]; combined analysis [TFRC (2.21), PGK2 (2.85), GAPDH (4.21)], respectively (Table 2).

To overcome different software program limitations, the stability of candidate reference genes was determined based on the consensus ranking for gene expression normalization in 7th and 18th-day embryos of the control broiler and improved Aseel. Our study identified the most stable genes and indicated that TFRC and PGK2 for the 7th and 18th-day embryos of the control broiler improved Aseel. Our observations further strengthen the necessity to analyze the stability of candidate reference genes as suitable references.

### Expression of muscle-related genes

The difference between fast and slow growth generally depends on the combination of environmental and genetic factors. The embryos collected in this experiment are under the same growth environment. In this experiment, the DEGs related to the main causes of growth and development differences mainly included muscle system processes, muscle tissue morphogenesis, muscle organ morphogenesis, etc. (Figure 4). The genes enriched by these entries are mostly muscle-related genes such as TNNC1, TNNT2, MYL3, MYH7, and FBXO32. The contraction of skeletal muscle-related genes is TNNC1, TNNT2, MYL3, and MYH7. In animals, skeletal muscle contraction affects growth traits like muscle protein, muscle fiber diameter, and muscle fiber density (Chen, 2012; Zhang, 2014). In the corpus callosum, the muscle is the most crucial component, and the sarcomere is composed of thick and thin muscles. Myosin molecules are mainly involved in thick filament formation, and the thin filament is a complex containing troponin, actin, and tropomyosin (Dong, 2017). The skeletal muscle contraction is mainly based on the relative sliding of thick and thin filaments (Li and Ren, 2007). For controlling the poultry muscle, according to previous reports, the troponin family gene is an important candidate gene (Chen, 2012; Chen L. et al, 2015; Zhao et al., 2018). In this experiment, the DEGs obtained related to the troponin family and essential components of skeletal muscle are troponin T type 3 (TNNT3), slow muscle troponin T (TNNT1), and troponin I type 1 (TNNI1). The myosin superfamily is a highly conserved family of proteins composed of a heavy chain, an alkaline light chain, and a light chain that are widely present in eukaryotic cells (Liu, 2013). In striated muscle, smooth muscle, and non-muscle, myosin is involved in myofilaments as a class II. The MYH gene family is a crucial subunit in the myosin class II molecule and encodes the myosin heavy chain (MYHC) (He and Gu, 2017). The studies revealed that MYH7 gene mutations could cause skeletal muscle disease or skeletal muscle disease with cardiomyopathy (Yuceyar et al., 2015; Feinstein-Linial et al., 2016). In the chicken breast muscle, the MYH7 gene expression is high, and it is a hypothesis that it has a regulatory role in muscle tissue growth and development (Zhang Z. et al., 2016). The MYL3 gene is primarily expressed in slow muscle and is a member of the myosin light chain (MYL) gene family (Zhang C. et al., 2016). Inhibition of myosin light chain gene expression in cell lines showed myoblast proliferation (Zhang et al., 2009). The chicken embryonic leg muscle proteome analysis found that MYL3 protein is closely related to the regulation of muscle contraction and the long non-coding RNAs Inc00037615 and Inc00037619 together regulate muscle development (Ouyang et al., 2017). In this experiment, the DEGs obtained from the myosin family are MYH7 and MYL3. In this study, five genes related to muscle contraction were screened, that is, TNNT3, TNNT1, TNNI1, MYL3, and MYH7. A transcriptome study showed that the expression of these five genes is high in improved Aseel slow growth compared with control broiler fast growth. Also, these genes are simultaneously present in two significantly enriched pathways, that is, adrenergic signaling in cardiomyocytes and cardiac muscle contraction. Perhaps as a result of the upregulation of these genes and the downregulation of Adrenergic signaling in cardiomyocytes and cardiac muscle contraction, the improved Aseel will grow more slowly than the control broiler. Previous studies showed that in animals under fasting, FBXO32 gene expression was significantly increased, muscles were degraded due to lack of food, and maybe it is associated with muscle atrophy (Bodine et al., 2001; Cleveland and Evenhuis, 2010). The FBXO32 gene expression was found in the chicken’s leg muscles, heart, and chest muscles and played an essential role in the 7th-week growth of chickens (Chen C. F. et al., 2015). In results of this transcriptome data showed that the expression of FBXO32 and FBXO7 genes was significantly lower in slow-growth improved Aseel than in the fast-growth control broiler, consistent with previous studies. The DEGs KEGG pathway enrichment analysis revealed significantly enriched adrenergic signaling in cardiomyocytes, cardiac muscle contraction, and tight junction signaling pathways. In cell junctions, the tight junction is an essential component, and it acts as a barrier for cells to pass ions and molecules, plasma membrane apical movement regulation, and basal proteins and lipids (Liu et al., 2012; Zhang et al., 2015). In all eukaryotic cells, a tight junction is recognized as the actin cytoskeleton, and it is involved in cell division, adhesion, movement, and phagocytosis and is also found in the chicken leg muscle transcriptome (Hartsock and Nelson, 2008; Xue et al., 2017). The muscle growth epigenetic transcriptional regulators are differentially regulated in 7EB, such as the protein arginine N-methyl transferase family (PRMT1 and PRMT3), histone lysine N-methyl transferases (EHMT1, and SETDB1), and SWI/SNF chromatin-remodeling enzymes (SmarcB1 and SmarcaA4).

### Muscle development and myostatin signaling

The muscle development and differentiation-related genes such as MYOD1, MYF6, MYF5, Myoz2, MAP2K6, MAP3K7, CAV1, CAV2, CAV3, HSP70, and NCF2 were differentially regulated in slow-growing improved Aseel then compared to fast-growing control broiler (Figure 4). In muscle differentiation, MYOD1 promotes muscle-specific gene expression and function together with MYF5 and MYOG (Akizawa et al., 2013). MYOD1, combined with transient placeholder protein, prevents the binding of other transcription factors to DNA and retains the inactive conformation of the DNA (Sartorelli et al., 1997). One of the critical functions of MYOD is to stop differentiated myocyte proliferation by enhancing the transcription of p21 and myogenin to remove cells from the cell cycle (Milewska et al., 2014). Altogether, upregulation of MYOD1 is involved in skeletal muscle phenotype establishment by regulation of precursor cell proliferation and promoting irreversible cell cycle arrest, facilitating differentiation and sarcomere assembly by activating sarcomeric and muscle-specific genes (Buckingham and Rigby, 2014). For this reason, transcriptome data shows downregulation of MYOD1 on the 7th day of an improved Aseel embryo; it may be due to this reason that muscle-specific gene proliferation is slow in improved Aseel. Myozenin is an α-actinin- and γ-filamin-binding protein of Z-line skeletal muscle that binds to calcineurin and is involved in skeletal muscle myocyte differentiation (Frey et al., 2000; Takada et al., 2001). Therefore, the upregulation of MYOZ2 and MYOZ3 genes in muscle tissues suggests that they are involved in muscle growth and development and directly influence meat quality. In addition, MYOZ1 and MYOZ2 genes expressed in mice significantly reduced calcineurin gene expression (Frey et al., 2004, 2008; Schulz and Yutzey, 2004). Our transcriptome study shows less expression of the MYOZ2 gene and high expression of the regulator of calcineurin one gene on the 7th day of improved Aseel embryo; maybe this is the region where muscle development is slow in improved Aseel. Mitogen-activated protein kinases are major components of pathways controlling embryogenesis, cell differentiation, cell proliferation, cell death, muscle development, and response to environmental stress (Errede et al., 1995; Gustin et al., 1998; Lewis et al., 1998; Davis, 2000). MAP2K6 and MAP3K7 activate MAP kinase and nuclear factor-kappa β (NFκB) and play an important role in its signal transduction pathway (Raingeaud et al., 1996; Ninomiya-Tsuji et al., 1999). A proteomic study predicted the expression of MAP2K1, MAP2K2, and MAP2K4, MAP4K4 genes, which may inhibit the low feed efficiency phenotype (Kong et al., 2016). In our transcriptome data, the mitogen-activated protein kinase family genes are upregulated in the 7th-day embryo of improved Aseel. Our results correlate with these results, which may be why the improved Aseel has less feed efficiency and slower muscle growth.

In this study, the expression of caveolin family genes like CAV1, CAV2, and CAV3 was downregulated in the 7th-day embryo of improved Aseel. In cell signaling, the caveolin genes act as sub-cellular structures by assisting the attribute of hormonal signals after binding hormones to the target receptor on the cell surface. CAV3 acts as a muscle-specific isoform for the caveolin protein, and mutations or different expressions of CAV3 can result in muscle myopathy (Biederer et al., 1998; Woodman et al., 2004). In pigs, CAV3 expression was upregulated during muscle hyperplasia, and it may be used as a genetic marker for meat production in swine (Zhu et al., 2006). In mice, the high or low expression of CAV3 made muscle cells more susceptible to oxidative stress and reduced survival through PI(3)K/Akt signaling (Smythe and Rando, 2006). In the low FE PedM broiler phenotype, the higher expression of CAV3 contributed to higher oxidative stress and enhanced muscle development (Bottje and Carstens, 2009). In high FE breast muscle, the CAV1 protein is involved in insulin signaling (Kong et al., 2016). Based on previous reports, downregulation of caveolin family genes in the 7th-day embryo of improved Aseel may reduce the improved Aseel muscle development. In high FE breast muscle, the up-regulation of HSP70 maintains muscle fiber integrity and enhances muscle regeneration and recovery from damage (Senf et al., 2013). HSP70 is also responsible for the correct folding and assembly of nuclear-encoded proteins, an essential chaperone for mitochondrial DNA-encoded proteins as components of the mitochondrial electron transport chain targeted for import into the mitochondria (Herrmann et al., 1994; Truscott et al., 2003). In low and high FE phenotypes, higher expression of HSP90 and HSPB2 was in response to oxidative stress, respectively (Bottje et al., 2012; Kong et al., 2016). Our transcriptome study correlated with previous studies, and we observed up-regulation of HSP70 and HSPB1 on the 7th day and downregulation of HSP90 on the 18th-day of improved Aseel embryo. The improved Aseel muscle may grow in this region more slowly compared to the control broiler. In NADPH oxidase 2 (NOX2), the NADPH/NADH oxidase is a critical component and is encoded by neutrophil cytosolic factor 2 (NCF2) (Ferreira and Laitano, 2016). In muscle, superoxide was generated by NOX2 in the sarcoplasmic reticulum, a major source of oxidative stress (Dikalov, 2011; Ferreira and Laitano, 2016). In neutrophils, NADPH generates superoxide during phagocytosis. The nuclear factor erythroid 2-like 2 (NFE2L2) is a downstream target for NOX2 and activates genes that contain an antioxidant response element in their promoter regions (Kobayashi et al., 2004, 2006). In high FE animals, it is predicted that NFE2L2 expression should be upregulated (Zhou et al., 2015; Kong et al., 2016). In a high FE commercial broiler, the up-regulation of NCF2 was associated with muscle remodeling and hypertrophy (Zhou et al., 2015). In our transcriptome data, NCF1, NOX1, and NOX3 were upregulated on the 7th-day, and NCF2 was downregulated on the 18th-day of the improved Aseel embryo. Maybe due to the differential expression of these genes, the improved Aseel is more resistant to oxidative stress, low FE, and slow growth.

Myostatin is a member of the tumor growth factor β (TGF-β) family and is known as growth/differentiation factor 8 (GDF-8) (McPherron et al., 1997; Lee and McPherron, 2001). In the myostatin (MSTN) signaling pathway, MSTN binds to its receptors ActIIA/ActIIB and activates ALK4 and ALK5 that phosphorylate Smad2/3, leading to its binding with Smad4 and translocation of the complex to the nucleus, and where it blocks the transcription of genes responsible for myogenesis (Lee et al., 2005; Elkina et al., 2011; Han and Mitch, 2011; Lee and Glass, 2011; Lassiter et al., 2019). Myostatin is solely expressed in skeletal muscle during embryogenesis to control the differentiation and proliferation of the myoblasts (McPherron et al., 1997). However, in the adult stage, it is expressed not only in skeletal muscle but also in other tissues like the heart, adipose tissue, and mammary gland (McPherron et al., 1997; Ji et al., 1998; Sharma et al., 1999; Morissette et al., 2006; Shyu et al., 2006; Allen et al., 2008). In turkey satellite cells, MSTN is a strong negative regulator for skeletal muscle growth, differentiation, and proliferation (McPherron et al., 1997; McFarland et al., 2006). The relation between MSTN and growth performance studies in broilers shows that MSTN is a polymeric gene in which different alleles can affect performance (Gu et al., 2004; Ye et al., 2007; Bhattacharya and Chatterjee, 2013). In the PedM broiler, the FE differences may be due to different haplotypes of the MSTN gene (Lassiter et al., 2019). Myostatin knockdown by RNAi shows muscle growth enhancement in transgenic sheep and chickens (Tripathi et al., 2012; Hu et al., 2013; Bhattacharya et al., 2019). In the present study, MSTN was differentially regulated in the 7th-day improved Aseel embryo. Maybe differential regulation of myostatin is needed for myoblast’s differentiation and proliferation in initial embryogenesis. Follistatin (FSTN) regulates the MSTN by inhibiting or limiting its activity. Follistatin-like 1 (FSTL1) is a glycoprotein and rich in cysteine (SPARC) family and comprises a secretion signal, a Follistatin and a Kazal-like domain, two EF-hand domains, and a von Willebrand factor type C domain (Sylva et al., 2013) (http://www.uniprot.org/uniprot/Q12841). In mice, FSTL1 is broadly expressed throughout the entire embryo and restricted to most of the tissues at the end of gestation, but in the adult mouse, it is highly expressed in the heart, lung, and subcutaneous white adipose tissue (Adams et al., 2007; Wu et al., 2010). In this study, FSTL1 was upregulated and follistatin/kazal downregulated on the 7th and 18th-day of improved Aseel embryo, respectively. Initial upregulation and later downregulation of FSTL1 may initiate muscle proliferation in the 7th-day and 18th-day embryo, slowing muscle development in improved Aseel. In humans, activin receptor type-1B (ACVR1B) or ALK4 is a protein that acts as a transducer of activin or activin-like ligand signals (Ten Dijke et al., 1993). ACVR1B forms a complex with ACVR2A/ACVR2B and goes on to recruit SMAD2/SMAD3 (Inman et al., 2002). In addition, ACVR1B transduces nodal, GDF-1, and Vg1 signals combined with other coreceptor molecules like protein cripto (Harrison et al., 2003). Transforming Growth Factor-β (TGFβ) is a key player in cell proliferation, differentiation, and apoptosis and TGFβ receptors are single-pass serine/threonine kinase receptors and can be eminent by their structural and functional properties (Dore et al., 1998). Due to their similar ligand-binding affinities, the transforming growth factor beta receptor I (TGFβR1)/ALK5 and TGFβR2 can be distinguished from each other by peptide mapping only. In mice, the TGFβ1 mRNA/protein has been present in cartilage, endochondral, membrane bone, and skin and plays a role in the growth and differentiation of these tissues (Dickinson et al., 1990). In the present study, activin A receptor type IB (ALK4) and transforming growth factor beta receptor II (TGFBR2) were upregulated, and transforming growth factor beta receptor I (TGFBR1/ALK5) was downregulated in the 7th-day embryo of improved Aseel. The differential expression of ALK4 and ALK5 may control the myostatin signaling pathway. The SMADs are important for regulating cell development, and growth and they have structurally similar proteins and are the main signal transducers for TGFβ receptors. The eight SMAD genes are distributed into three sub-types of SMADs; they are R-SMADs, Co-SMADs, and I-SMADs (Derynck et al., 1998; Massague et al., 2005). The R-SMADs consist of Smad1, Smad2, Smad3, Smad5, and Smad8/9 and are involved in direct signaling from the TGFβ receptor (Wu et al., 2001; Massague, 2012). The Co-SMADs consist of only SMAD4 and work jointly with R-SMADs to recruit co-regulators to the complex (Shi et al., 1997). R/Co-SMADs are primarily located in the cytoplasm, following TGFβ signaling, and later accumulate in the nucleus, where they can bind to DNA and regulate transcription. I-SMADs consist of SMAD6 and SMAD7 and are predominantly found in the nucleus, where they can act as direct transcriptional regulators. SMAD6 is specifically associated with BMP signaling and SMAD7 is a TGFβ signal inhibitor and suppresses the activity of R-SMADs (Itoh et al., 2001; Macias et al., 2015; Yan et al., 2016). In the present transcriptome study, the SMAD family member 1 (SMAD1), SMAD specific E3 ubiquitin protein ligase 2, SMAD family member 3 (SMAD3), SMAD family member 5 (SMAD5), and TGF-βsignal pathway antagonist Smad7 (SMAD7B) upregulated on the 7th day of the improved Aseel embryo, and they may control the myostatin signaling pathway in the improved Aseel embryonic stage. Summarizes the initial steps in the MSTN signaling pathway in the present study that would potentially exert a negative effect on muscle differentiation and proliferation in the slow-growing improved Aseel.

### Energy sensing, fatty acid metabolism, and embryo development

In humans and animals, the adenosine monophosphate-activated protein kinase (AMPK) gene regulates diverse biological functions (Hardie et al., 1998). The mammalian 5′ AMPK gene has two α subunits that is, AMPKα1 and AMPKα2 that are encoded by Prkaa1 and Prkaa2 genes, respectively. The knockout mouse clearly demonstrated that AMPKα2 controls homeostasis in skeletal muscle (Viollet et al., 2003a,b). Also observed was a reduction in fiber numbers (∼25%) and sizes (∼20%) in the soleus muscle of AMPKα1 knockout mice (Fu et al., 2013). However, in AMPKα2 knockout mice, both fiber size and muscle mass were significantly increased, while the muscle fiber number remained similar to WT animals. The muscle mass reduced and increased differentially expressed alternative polyadenylation sites (DE-APSs) were downregulated in AMPKα1 knockout mice but upregulated in AMPKα2 knockout mice, respectively (Zhang et al., 2018). The five genes, that is, carbonic anhydrase 3 (Car3), myosin light chain kinase family, member 4 (Mylk4), nebulin (Neb), obscurin (Obscn), and phosphofructokinase, muscle (Pfm) are utilized by different APSs and show potential effects on muscle function (Zhang et al., 2018). The high FE phenotype birds show up-regulation of both AMPKα1 and AMPKα2 (Bottje et al., 2012). In low energy level conditions, AMPK gene expression increases ATP production by inhibiting the ATP-consuming pathways like fatty acid synthesis, protein synthesis, and gluconeogenesis and stimulating the ATP-producing pathways like mitochondrial biogenesis and oxidative phosphorylation, glycolysis, and lipolysis (Zhou et al., 2001; Hardie et al., 2003; Carling, 2004). In the present study, the AMPK genes like 5′-AMP-activated protein kinase gamma-1 non-catalytic subunit variant 1 (PRKAG1), protein kinase cAMP-dependent regulatory type I alpha (tissue specific extinguisher 1) (PRKAR1A), protein kinase AMP-activated beta 2 non-catalytic subunit (PRKAB2), protein kinase AMP-activated gamma 2 non-catalytic subunit (PRKAG2), carbonic anhydrase XIII, myosin light chain kinase (MYLK), atrial/embryonic alkali myosin light chain, are downregulated in the 7th-day of improved Aseel embryo, may be due to the downregulation of energy-producing pathways, the improved Aseel muscle will grow slowly compared to control broiler. Curiously, creatine kinase (muscle isoform, CK-M) and creatine kinase (brain isoform, CK-B) were upregulated in high and low FE phenotypes, respectively (Kong et al., 2016; Bottje et al., 2017a,b). The reason for this discrepancy may be that the high FE phenotype of broiler breast muscle has enhanced capabilities for mitochondrial oxidative phosphorylation as well as creatine and phosphorylated creatine shuttle in and out of mitochondria (Bottje et al., 2017b). In the present study, the creatine kinase muscle (CKM), creatine kinase mitochondrial 1A (CKMT1A), creatine kinase brain (CKB), and creatine kinase mitochondrial 2 (sarcomeric) (CKMT2) genes were upregulated in the 7th-day embryo of improved Aseel. In skeletal muscle, nitric oxide is synthesized by nitric oxide synthases, and it is regulated by key homeostatic mechanisms like mitochondrial bioenergetics, network remodeling, mitochondrial unfolded protein response (UPRmt), and autophagy (Figures 4, 5). In mice, nitric oxide synthase deficiency inhibits the Akt-mammalian target of the rapamycin pathway and dysregulates the Akt-FoxO3-mitochondrial E3 ubiquitin-protein ligase 1 (Mul-1) axis (De Palma et al., 2014). Thus, mitochondrial biogenesis and body energy balance were controlled by the nitric oxide-cGMP-dependent pathway (Nisoli et al., 2003). In detail, the inhibition of nNOS/NO/cGMP-dependent protein kinases enhanced the FoxO3 transcriptional activity and increased the Mul-1 expression. The absence of the nitric oxide synthases significantly impaired muscle fiber growth with muscle force, decreased resistance to fatigue, and degeneration/damage post-exercise. In our study, nitric oxide synthase 2 was upregulated, and cGMP-dependent protein kinase type I and FOXO1 were downregulated on the 7th-day of the improved Aseel embryo, maybe that this is the region where the improved Aseel muscle strength was high, and they are more energetic compared to the control broiler.

The comparative muscle transcriptome analysis between high and low pH chickens showed that most of the glycolysis pathway genes are upregulated in the lower pH chicken (Marziano et al., 2017). The previous study shows that Aseel and broiler chicken’s meat do not have any significant pH variation, but the heavier bird’s meat had a significantly higher pH (Rajkumar et al., 2016). In this study, glycolysis metabolism-related genes are differentially regulated on the 7th-day of an improved Aseel embryo. The glucose-6-phosphate isomerase, fructose bisphosphate aldolase, phosphoglycerate kinase, and enolase were downregulated, and GAPDH, phosphoglycerate mutase, and pyruvate kinase were upregulated in the 7th-day embryo of improved Aseel. Fructose bisphosphate aldolase is a key enzyme in glycolysis as well as gluconeogenesis and is involved in the stress-response pathway during hypoxia (Beauclercq et al., 2017). The high pH chickens have increased oxidative stress, maybe the higher expression of fructose bisphosphate aldolase is linked to its function in the stress-response pathway rather than to its role in ATP biosynthesis (Beauclercq et al., 2016). In our study, downregulation of fructose bisphosphate aldolase enhanced the ATP synthesis, maybe this is the region where improved Aseel birds have more energy than control broilers. Noteworthy, the up-regulation of glycolysis pathway genes increases the pyruvate levels and enters the citric acid cycle, and thus, higher levels of ATP are produced in improved Aseel. The protein phosphatase-1 regulatory subunit 3A (PPP1R3A) binds glycogen with high affinity, activates glycogen synthase (GYS), and inhibits glycogen phosphorylase kinase (PHK) by dephosphorylation through the protein phosphatase-1 catalytic (PPP1C) subunit. In this study, the glycogen metabolism genes that is, protein phosphatase-1 regulatory subunit 2 (PPP1R2), glycogenin 1, glycogen phosphorylase, and protein phosphatase-1 catalytic subunit beta (PPP1CB) were downregulated in the 7th-day embryo of improved Aseel, maybe this is the region that the improved Aseel muscle has more glycogen. The AMP-activated protein kinase (AMPK) complex is another key regulator of glycogen turnover, and it consists of one α catalytic and two non-catalytic subunits, β, and γ. The β subunit binds to glycogen along with α and γ subunits and forms a heterotrimeric AMPK complex. In the muscle cell, the γ subunits of the AMPK complex act as energy sensors and bind to AMP and ATP (Cheung et al., 2000). In our study, AMP-activated protein kinase beta 2 non-catalytic subunit (PRKAB2), cAMP-dependent protein kinase regulatory type I alpha (tissue-specific extinguisher 1) (PRKAR1A), AMP-activated protein kinase gamma 2 non-catalytic subunit (PRKAG2) were downregulated, and 5′-AMP-activated protein kinase gamma-1 non-catalytic subunit variant 1 (PRKAG1), AMP-activated protein kinase gamma three non-catalytic subunit (PRKAG3), AMP-activated protein kinase alpha 2 catalytic subunit (PRKAA2) were upregulated. The downregulation of β subunits and up-regulation of α and γ subunits may balance the glycogen accumulation and increase the ATP molecules for energy production in the improved Aseel muscle, maybe this is the region where the improved Aseel is stronger than the control broiler. Apart from these, several other genes indirectly influence glycogen storage in muscle. The phosphodiesterase 3B (PDE3B) gene is activated by insulin and induces antiglycogenolytic effects, and the mitochondrial creatine kinase (CKMT2) transfers the high-energy phosphate from mitochondria to creatine. In our study, phosphodiesterase 3A (PDE3A), phosphodiesterase 4D, phosphodiesterase 8A, mitochondrial creatine kinase 2 (CKMT2), mitochondrial creatine kinase 1A (CKMT1A), and mitochondrial creatine muscle (CKM) were upregulated in the 7th-day of embryo improved Aseel, maybe this is the region where the higher expression of these genes in muscle means improved Aseel birds are more energetic compared to control broiler. To produce energy and compensate for the lack of energy due to carbohydrates and glycolysis, the high pH chicken’s muscle asks for more intense oxidative pathways, such as lipid β-oxidation and ketogenic amino acid degradation (Beauclercq et al., 2016). In the high pH muscle line, the 3-hydroxymethyl-3-methylglutaryl-CoA lyase (HMGCL) catalysis the final step of leucine metabolism and ketone metabolism, acetyl-CoA acetyltransferase-2 (ACAT2) involved in β-oxidation or degradation of ketogenic amino acids, and nudix hydrolases (NUDT7, NUDT12, NUDT19) hydrolyse a nucleoside di and triphosphates, dinucleoside and diphosphoinositol polyphosphates, nucleotide sugars and RNA caps, were upregulated. In the present study, 3-hydroxymethyl-3-methylglutaryl-CoA lyase like-1 (HMGCLL1), acetyl-CoA acetyltransferase 2 (ACAT2), nudix type motif 7 (NUDT7), and nudix type motif 21 (NUDT21) were downregulated, and carnitine/palmitoyl-transferase 1 (CPT1) was upregulated in the 7th-day embryo of improved Aseel (Figure 6). They may regulate the β-oxidation in peroxisomes as well as mitochondria; this is the region may be fats required for initial embryo development and excess fats involved in β-oxidation and finally provide the energy for embryo development.

### Protein synthesis

To promote cell growth, the mTORC1 complex increases protein synthesis, lipid metabolism, and autophagy inhibition and regulates the transcription of several genes (Laplante and Sabatini, 2013). In the high FE phenotype, the cDNA microarray data shows a higher expression of mTORC1 (Schiaffino et al., 2013; Bottje et al., 2014). The mTORC1 complex has two major components, that is, mTOR and RAPTOR (Kim et al., 2002). RAPTOR and mTOR were up and downregulated in high and low FE birds, respectively, and the up-regulation of RAPTOR could have a positive effect on protein synthesis (Kim et al., 2002). In the low FE phenotype, PRKAR1A and GLUT-8 were upregulated. p70S6k and eukaryotic translation initiation factor 4E (EIF4E) are the key downstream targets for mTORC1 and are involved in enhancing protein synthesis. In low FE birds, the expression of p70S6k was higher (Bigot et al., 2003). The muscle tissue of RNAseq transcriptomic data showed higher expression of eukaryotic initiation, elongation, and translation genes in high FE compared to the low FE PedM phenotype (Bottje et al., 2017a,c). In the present study, the late endosomal/lysosomal adaptor MAPK and MTOR activator 3 (LAMTOR3), protein kinase, cAMP-dependent, regulatory, type I, alpha (tissue specific extinguisher 1) (PRKAR1A), solute carrier family 2 (facilitated glucose transporter) member 8 (SLC2A8)/GLUT8/GLUTX1, solute carrier family 2 (facilitated glucose transporter), member 3 (SLC2A3), ribosomal protein S6 kinase, 90kDa, polypeptide 1 (RPS6KA1), ribosomal protein S6 kinase, 90kDa, polypeptide 3, ribosomal protein S6 kinase, 52kDa, polypeptide 1, ribosomal protein S6 kinase, 90kDa, polypeptide 6, ribosomal protein S6 kinase-like 1, KIAA1328, KIAA1324, and eukaryotic translation initiation factor 4E binding protein one gene were downregulated in the 7th-day embryo of improved Aseel compared to control broiler (Figure 4). Protein synthesis for muscle growth may be lower in improved Aseel compared to control broiler due to downregulation of mTORC1 complex and ribosomal machinery genes.

### Insulin signaling

In chickens, SHC1 is only activated by nutritional changes, suggesting that insulin signaling in chickens has a tissue-specific manner (Dupont et al., 1998a,b). When insulin binds to the insulin receptor, both IRS-1 and SHC1 are activated by a phosphoinositide-3 kinase (PI3K) mediated tyrosine phosphorylation activity (Bottje et al., 2012). The skeletal myoblast is mainly differentiated by two key modulators, that is, insulin-like growth factor 1 (IGF1) and fibroblast growth factor 2 (FGF2) (Florini and Magri, 1989; Florini et al., 1991a). In L6 and C2C12 myoblasts, a high concentration of IGFs reduces their differentiation, whereas a low concentration enhances their differentiation (Florini et al., 1991b, 1986). The IGF binding proteins (IGFBP-1 to IGFBP-6) have highly conserved regions and bind with high affinity to IGF-1 and IGF-2. In the extracellular matrix, IGFBP3 may regulate the interaction of IGFs and it is present in rat soleus muscle (type I muscle fiber) (Stewart and Rotwein, 1996; Spangenburg et al., 2003). In humans, IGFBP3 also plays a role in myoblast differentiation (Foulstone et al., 2003). The fibroblast growth factor 2 (FGF2), transforming growth factor beta (TGFb), and oncogenic Ras also inhibit skeletal myoblast differentiation (Florini and Magri, 1989). The 23A2 myoblast cell lines show the inhibition of 23A2 myoblasts differentiation by IGF1 and FGF2 by stimulating the signaling through mitogen-activated protein kinase (MAPK) kinase (MEK) to MAPK (Weyman and Wolfman, 1998). In our study, IGF2, FGF2, IGFBP2, IGF2BP1, IRS-1, IRS-2, INSIG1, PIK3CA, PIK3CD, TGFb, and Ras oncogene family genes are upregulated in the 7th-day embryo of improved Aseel (Figure 7). Maybe this is also one of the regions for improved Aseel muscle which differentiates slowly when compared to the control broiler.

### Expression of plumage development genes

In vertebrate coloration, melanin pigmentation is an important component and is regulated by strong genetic control (Roulin, 2004; Plonka et al., 2009). In chickens, plumage coloration development is extremely complex and can be classified as structural or pigment-based (Lee et al., 2016). For animal coloration, melanin is a common component, synthesized in melanocytes and deposited in various organs as granules (Muroya et al., 2000). Different pigment patterns are formed based on the presence of melanocytes modulating, arranging, or differentiation and associated with a series of functional genes (Lin et al., 2013; Yu et al., 2018). The melanogenesis genes such as HOX, CHAC1, GPX3, BMP5, PITX2, RGN, MITF, TYR, KIT, OCA2, ASIP, MCIR, KITLG, IRF4, SLC24A4, SLC45A2, EDN, TYRP1, and TYRP2 are involved in melanin pigmentation (Zhang et al., 2015a,b; Duffy et al., 2010; Nan et al., 2009; Sulem et al., 2007). The homeobox (HOX) genes are transcription factors and involved in skin appendage development, including hair follicles and feathers (Chuong et al., 1990; Kanzler et al., 1994; Stelnicki et al., 1998; Godwin and Capecchi, 1999; Packer et al., 2000; Awgulewitsch, 2003). In black-bone chickens, four HOX genes, that is, HOXB9, HOXC8, HOXA9, and HOXC9, were identified for melanin pigmentation (Yu et al., 2018). Wnt signaling is essential for skin organogenesis and its appendages like hairs, feathers, and scales, melanocyte development, and differentiation (Yamaguchi et al., 2004, 2007; Widelitz, 2008; Cho et al., 2009). HOXB9 is identified as a target gene for Wnt signaling and HOXC8 is expressed in the first stage of feather morphogenesis like dorsal dermal and epidermal cells (Kanzler et al., 1997; Nguyen et al., 2009). In this study, HOXA2, HOXA9, HOXB3, HOXB5, HOXB7, HOXB8, HOXB9, HOXC11, HOXD1, and HOXD3 are upregulated in the 7th-day improved Aseel embryo, maybe this is the region the where improved Aseel plumage has multiple colors.

In animals, melanogenesis is regulated by GSH and it is closely associated with melanin deposition in the skin of humans and other mammals (Halprin and Ohkawara, 1966; Meister, 1983; Ito et al., 1985; Meyskens et al., 2001; Galvan and Alonso-Alvarez, 2008). The low and high levels of GSH indicate eumelanin-type pigmentation and phaeomelanin-producing melanocytes found in the skin, respectively (Benedetto et al., 1981). Two feather melanin pigmentation genes were identified in black-bone chickens, such as ChaC glutathione-specific gamma-glutamylcyclotransferase 1 (CHAC1) and glutathione peroxidase 3 (GPX3) (Yu et al., 2018). The CHAC1 cleavage of GSH into 5-oxoproline and Cys-Gly dipeptide and GSH over-expressed mammalian cells causes GSH depletion (Kumar et al., 2012; Crawford et al., 2015). Hence, CHAC1 expression is associated with GSH metabolism and plays an important role in the melanogenesis process. In eumelanin and phaeomelanin synthesis, the hydrogen peroxide is reduced by GSH-dependent peroxidase and GPX3 belongs to the GSH peroxidase family and catalyzes the GSH to glutathione disulphide (GSSG) (Benathan, 1997; Meyskens et al., 2001). The melanoma cell’s pigmentation is regulated by GSH levels, glutathione peroxidase, and glutathione reductase, suggesting that GSH-mediated redox processes play an important role in melanogenesis regulation (Benathan et al., 1999). Hence, the expression of GPX3 plays an active role in chicken feather melanogenesis. In this study, CHAC1, CHAC2, gamma-glutamylcyclotransferase (GGCT), and GPX8 were downregulated on the 7th-day of the improved Aseel embryo, maybe this is the region the improved Aseel has multiple colors on their feathers. For plumage melanogenesis in black-bone chickens, two pathways were identified: that is, the TGF-β signaling pathway, and ascorbate and aldarate metabolism (Yu et al., 2018). TGF-β regulates the proliferation and synthesis of melanin in chicken retinal pigment epithelial cells (Kishi et al., 2001). The BMP5 and PITX2 genes are involved in the TGF-β signaling pathway and play a role in the synthesis of chicken melanin, and BMP5 and PITX2 were found to be highly expressed in white and black feather bulbs, respectively (Kishi et al., 2001). The BMP3 gene was highly expressed in embryonic and post-embryonic stages of the control layer when compared to broiler chicken, and the BMP4 gene was differentially expressed in juvenile stages of broiler and layer chicken, respectively (Divya et al., 2018a,b). The regucalcin (RGN) is a calcium-binding protein involved in the ascorbate and aldarate metabolism pathways and plays a crucial role in intracellular calcium homeostasis maintenance (Moisa et al., 2013). In this study, transforming growth factor beta receptor II (TGFBR2), BMP1, BMP1A, BMP4, BMP7, BMPR1A, BMPR2, and PITX3 were upregulated and RGN was downregulated on the 7th-day embryo of improved Aseel, maybe this is the region the improved Aseel plumage has multiple colors.

 melanin synthesis, TYR is a rate-limiting enzyme and is involved in different oxidative steps (Parvez et al., 2006; Olivares and Solano, 2009). In black vs. white skin chickens, the TYR is highly expressed and it is consistent with sheep coat color studies (Norris and Whan, 2008; Fan et al., 2013; Zhang J. et al., 2015). In black-coated vs. white-coated sheep, the TYRP1 gene was highly expressed (Fan et al., 2013). KIT is a receptor tyrosine kinase, the mutation in KIT showed piebaldism and auburn hair color in humans, and it plays an important role in UVB-induced melanogenesis in the *epidermis*, and inhibition of KIT expression may result in the lightening of human skin color (Yang Y. J. et al., 2013; Yamada et al., 2013). In black-skinned chickens, KIT is highly expressed, and black skin color is due to increased melanin compared to white skin color (Zhang J. et al., 2015). In melanocyte development, microphthalmia-associated transcription factor (MITF) plays a role, and mutations in the MITF gene are responsible for Japanese quail and chicken plumage color and it is supported by alternative splicing of the MITF gene in the skin of sheep (Minvielle et al., 2010; Saravanperumal et al., 2014). In ducks, TYR and MITF expression may involve the formation of black and white plumage (Li et al., 2012). Melanocortin-1 receptor (MC1R) binds to melanocyte stimulating hormone (MSH) to initiate the melanogenesis cascade and regulates mammalian skin pigmentation and hair color (Roberts et al., 2006; Schaffler et al., 2006; Solano et al., 2006; Lalueza-Fox et al., 2007). The agouti signaling protein (ASIP) is responsible for the skin color of both white and black-coated sheep, and mutations in ASIP cause black and tan pigmentation phenotypes in pigs (Drogemuller et al., 2006; Norris and Whan, 2008). The ASIP binds to MC1R and reduces the MITF and TYR gene expression, and finally, the phaeomelanin will be reduced in epidermal tissues. In black-skinned chickens, the expression of ASIP is higher than compared to white-skinned chickens, and it can suppress the MC1R gene expression in black-skinned chickens (Zhang J. et al., 2015). Oculocutaneous albinism type 2 (OCA2) is a common skin pigmentation disorder caused by a mutation in the OCA gene. In black chickens, OCA2 was upregulated and it may be related to black skin color (Zhang J. et al., 2015). In chickens, the endothelins (EDN1, EDN2, and EDN3) and their receptors (EDNRA, EDNRB, and EDNRB2) are involved in the regulation of pigmentation and plumage (Liu et al., 2019). The expression of EDNRB2 was significantly different between adult black and non-black chickens (Dorshorst et al., 2011). In this study, TYRP1, KITLG, MITF, MC1R, AGRP, YRK, and P56LCK were upregulated and EDNRA and EDN1 were downregulated on the 7th-day of the improved Aseel embryo, and this is the region where the improved Aseel plumage has multiple colors.

### Expression of genes related to egg production

In chickens, the reproductive system is regulated by hypothalamic–pituitary–ovarian (HPO) axis hormones, while ovulation, the GnRH-I triggers the pituitary gland to release FSH and LH, and stimulates the secretion of estradiol and progesterone in the ovary (Bain et al., 2016). Several reproductive hormone regulation genes were identified between high and low egg production chickens, such as HADH, HMGCR, RAB11FIP1, and FAM3D (Mishra et al., 2020). HMGCR, HMGCS1, NFKB1, VAV3, SOS1, IL1R1, MEF2C, and STK3 were highly expressed in the pituitary gland, as were lipid metabolic processes, prolactin, and MAPK signaling pathway genes. In chickens, the anterior pituitary gland synthesizes and releases prolactin and is involved in reproduction, laying eggs, metabolism, development, and hypothalamic–pituitary–gonadal axis regulation (Talbot et al., 1991; Reddy et al., 2002). In chicken, the HMGCR gene variants (G-789-A, C-937-G, and A-2316-C) and high and low concentrations of VLDL showed higher and lower egg production, respectively (Han et al., 2014). In laying chickens, the APOB is a primary organizing protein for chylomicrons and VLDL and is responsible for the transport of lipoprotein, circulating in the plasma and stored in the oocytes to form an egg yolk (Deeley et al., 1985; Nys and Guyot, 2011). In our study, GNRHR, HADHB, HMGCS1, HMGCS2, RAB11FIP2, RAB11FIP3, RAB11FIP4, NFKB2, VAV2, SOS2, MEF2D, STK3, PRL, PRLR, PRLH, and PRLHR2 genes were upregulated, and FSHR, VAV3, IL1RL1, and IL1RAPL2 genes were downregulated, and family with sequence similarity genes and apolipoprotein B were differentially regulated in the 7th-day embryo of improved Aseel. Maybe this is the region where the improved Aseel has less egg production than the commercial chicken. In avian species, the genes SPP1, BPIFB3, and EDIL3 are mainly involved in egg and oviposition, development of the reproduction system, and vesicle-mediated eggshell calcication, respectively (Jeong et al., 2012; Dong et al., 2019; Stapane et al., 2019; Yang et al., 2019). In this study, secreted phosphoprotein 1 (SPP1) and secreted phosphoprotein 2 (SPP2) genes are downregulated, and EDIL3 is upregulated on the 7th-day of an improved Aseel embryo, due to this, egg and oviposition are less, and eggshell calcication is more in improved Aseel. In nandan-yao chickens, FN1, FGF7, SOX2, ALDOB, and HSPA2 genes are expressed in the ovary, and UQCRH, COX5A, FN1, TGFB, and ACTN1 genes are expressed in the uterus and involved in egg production (Sun et al., 2021). In this study, FN1, FNDC3A, FGFR1, FGFR3, FRS3, FRS2, FGFR2, FGFRL1, FGF8, FGF18, FGF3, FGF12, SOX2, SOX3, SOX4, SOX5, SOX7, SOX8, SOX9, SOX11, SOX17, HSPA2, HSPA4, COX1, COX2, COX3, TGFB4, and ACTN1 were upregulated and ALDOB, HSPA5, HSPA8, HSPA9, HSP12A, UQCRFS1, UQCRB, were downregulated in the 7th-day improved Aseel embryo. The differential expression of the ovary and uterus-related genes is differentially expressed on the 7th-day of an improved Aseel embryo, due to this region, the egg production is less in improved Aseel. The DEGs related to the pituitary gland in high and low egg production chickens are mainly involved in mTOR and Jak-STAT signaling pathways (Mishra et al., 2020). In mice, the mTOR signaling pathway will regulate granulosa cell proliferation and differentiation (Yaba and Demir, 2012). In this study, the mTOR and Jak-STAT signaling pathways were upregulated in the 7th-day improved Aseel embryo.

In high egg production chickens, several embryonic development genes are upregulated, such as GDNF, HOXD9, MEF2C, STK3, CLRN1, IRX5, LBX1, CSNK1A1, LGR5, PRDM15, and DAB2IP (Mishra et al., 2020). In this study, the GDNF, HOXA2, HOXB3, HOXB5, HOXB7, HOXB8, MEF2D, STK3, STK16, STK25, STK32B, IRX1, IRX2, IRX5, IRX6, LBX1, LBX3, PACSIN2, RGR, PRDM4, PRDM8, and DAB2IP genes are upregulated on the 7th-day of the embryo, these genes are involved in embryo development. In the ovary, the tryptophan metabolism and PI3K-Akt signaling pathways were enriched, and they are important for egg production (Mishra et al., 2020). In stressful conditions, peripheral and brain tryptophan levels can be altered by stimulating the immune system and activating the hypothalamic–pituitary–adrenal axis (Miura et al., 2008; Birkl et al., 2019). In this study, tryptophan metabolism was downregulated on the 7th-day of the embryo and upregulated on the 18th-day thigh muscle, this is the region where the improved Aseel has less egg production. In high egg production, the hypothalamus genes are highly expressed, such as EXFABP, SNRNP25, FAM114A1, and SIX1 (Mishra et al., 2020). In the hypothalamus, nerve growth factor response, lipid metabolism, and canonical Wnt signaling pathway genes were highly expressed, that is, SIX1, RPS15, and IGFBP7, thus playing a role in chicken egg production. In laying hens, the dietary corticosterone treatment shows low levels of extracellular fatty acid-binding protein (EXFABP) and suggests that the egg white protein’s synthesis and secretion may be affected by environmental stress (Kim and Choi, 2014). Many studies have found that ovarian follicular development is stimulated by IGFBPs and plays a role in the ovary’s FSH action (Zhou et al., 1997; Mazerbourg et al., 2003). In chicken adipose tissue, the lipid metabolism gene like insulin-like growth factor binding protein 7 (IGFBP7) was highly expressed and it was correlated with egg production (Nagaraja et al., 2000; Wang et al., 2007). In this study, FAM114A, FAM116A, FAM116B, FAM117A, FAM117B, SIX1, SIX2, RPS13, RPS24, IGFBP2, IGFBP3, and IGFBP5 genes are upregulated and FABP1, FABP2, FABP3, FABP5, SNRPB, SNRPB2, IGFBP1, and IGFBP7 genes are downregulated in the 7th-day embryo of improved Aseel, these differentially expressed genes may cause less egg production in improved Aseel (Figure 7). The cuticle or organic matrix of the eggshell-related genes that is, MEPE, BPIFB3, RARRES1, and WAP are highly expressed in oviposition (Mann et al., 2006; Rose-Martel et al., 2012; Bain et al., 2013). In this study, RARA, RARB, POSTN, CDH4, CDH13, CDH8, CDH11, and CDH20 were upregulated, and RARRES1, and CDH1, were downregulated in the 7th-day improved Aseel embryo. Due to the differential expression of these genes, maybe the improved Aseel eggshell thickness is more than commercial laying eggs.

The mitochondrial oxidative phosphorylation, active transport, and energy metabolism related genes such as NADH dehydrogenase, ND4, ND1, ND2, ND5, ACTB, GAPDH, ATP6, and ATP1A are required for a large amount of energy and active secretion of proteins and minerals (Bar, 2009). A recent report shows differential expression of these genes like MEPE, COX1, COX3, COX2, BPIFB3, Cytochrome b, ATP6, ND5, ATP1A1, ND4, ND2, EIF4A2, UBB, Novel mitochondrial gene, IGLL1, HSPA8, RASD1 in the GNRH1 vs. AVT study (Pertinez et al., 2020). In this study, ND1, ND2, ND3, ND4, ND4L, ND5, ND6, GAPDH, ATP6, ATP1A2, COX1, COX2, COX3, and CYTB were upregulated, and NDUFAF1, NDUFA4, NDUFA5, NDUFA9, NDUFA10, NDUFAF4, ATP1A1, EIF4A2, COX15, COX19, COX20, CYB5A, CYB5B, and CYB5R2 were downregulated in the 7th-day of the improved Aseel embryo. Most of the energy metabolism genes are upregulated and maybe the region improved Aseel is stronger than commercial birds.

## Conclusion

**FIGURE 8:**
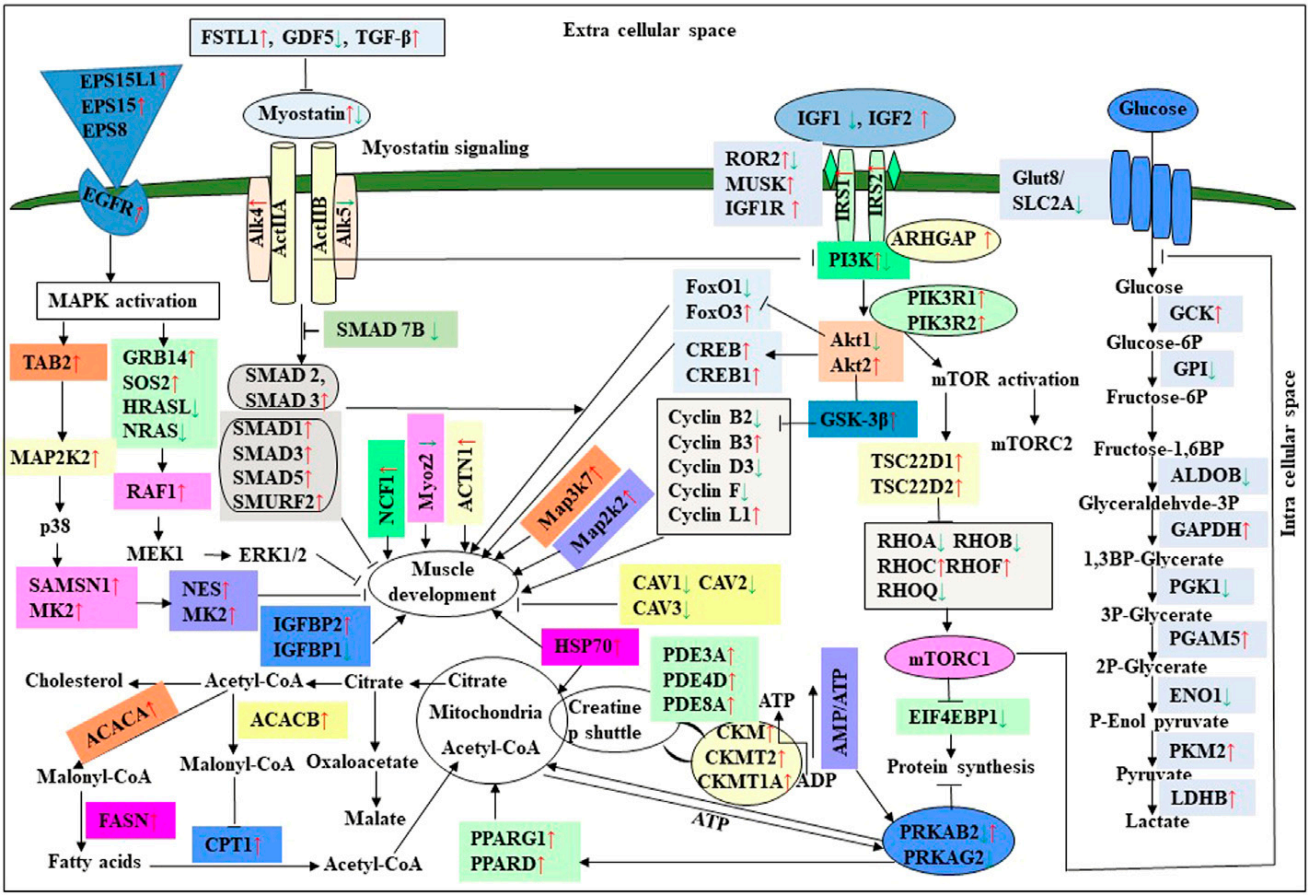
Diagrammatic representation of DEGs analysis in the 7^th^-day embryo and 18^th^-day thigh muscle of Aseel as compared to respective controls of the control broiler. The red and green arrows represent the up and downregulated genes (*p* < 0.05), respectively. → the activation of the process, ┤inhibition of the process. 1. Canonical pathway of Smad activation. Myostatin binds to ActRIIB and induces its assembly with activin type I receptor, subsequent phosphorylation of Smad2/3 leads to its binding with Smad4 and translocation of the complex to the nucleus where it blocks the transcription of genes responsible for the myogenesis. Smad6 and Smad7 compete for the binding with activin type I receptor, and Smad7 can also prevent the formation of the Smad2/3 and Smad4 complex. 2. MAPK activation. The activation of MAPKs is mediated *via* myostatin using different pathways: TAK-1/MAPKK for p38 MAPK or Ras/Raf/MEK1 for ERK1/2. It leads to the blockade of genes responsible for myogenesis. 3. Inhibition of Akt signaling. Akt phosphorylation occurs in the response to insulin and IGF-1. In a normal case, active Akt induces an mTOR signal leading to protein synthesis while inhibiting FoxO by phosphorylation. In pathological conditions, dephosphorylated Akt does not inhibit FoxO. It leads to the accumulation of FoxO in the nucleus where it binds to the DNA and induces the transcription of E3 ubiquitin ligases MURF-1 and Atrogin-1. Smad3 and Smad4 possibly participate in FoxO signaling.

The comparative transcriptome study between slow-growth improved Aseel and fast-growth control broiler revealed the DEGs and their significantly enriched pathways in slow-growth improved Aseel, which inferred that they play an important role in regulating the growth and development of improved Aseel. The transcriptome data provides a theoretical basis for improving the performance of the slow growth improved Aseel as well as how to control the growth performance of fast grown control broiler chickens and provides reference data for revealing the molecular mechanism of slow growth improved Aseel as well as fast growth control broiler chickens. In this study, the mechanistic picture of gene expression data (Figure 8) shows the embryo development, muscle development, egg production, plumage development, and energy production in improved Aseel would be fostered by a combination of 1) differential regulation of MSTN, activin-like kinasesn and upregulation of SMADs, expect SMAD7 in the myostatin signaling pathway, combined with downregulation of caveolin’s (CAV1, CAV2, and CAV3) and differential regulation of insulin-like growth factor binding proteins; 2) upregulation of HSP70, NCF1, and Map2k2 and downregulation of MYOD1 and MYOZ2; 3) upregulation of fatty acid synthesis and β-oxidation genes (ACACA, ACACB, FASN, and CPT1); 4) differential MAPK signaling pathway genes (MAP2K2, MKA, NES, SAMSN1, SOS2, and TAB2); 5) differential regulation of Jak-STAT, mTOR, and TGF-β signaling pathway genes (IGF1, IGF2, IRS1, IRS2, PI3K, Akt1, Akt2, FoxO1, FoxO3, TSC22D1, TSC22D2, RHOA, RHOB, RHOC, RHOF, RHOQ, and EIF4EBP1); 6) differential regulation of mitochondrial genes (ND1, ND2, ND3, ND4, ND4L, ND5, ND6, ATP6, ATP1A2, COX1, COX2, COX3, CYTB NDUFAF1, NDUFA4, NDUFA5, NDUFA9, NDUFA10, NDUFAF4, ATP1A1, EIF4A2, COX15, COX19, COX20, CYB5A, and CYB5B); 7) differential regulation of glycolysis/gluconeogenesis genes (GCK, GPI, ALDOB, GAPDH, PGK1, PGAM5, ENO1, PKM2, and LDHB).

## Data Availability

The datasets presented in this study can be found in online repositories. The names of the repository/repositories and accession number(s) can be found in the article/Supplementary Material.
