# Supplementary material for: Embryonic transcriptome unravels mechanisms and pathways underlying embryonic development with respect to muscle growth, egg production, and plumage formation in native and broiler chickens
Source: Front Genet. 2022 Oct 14;13:990849. doi: 10.3389/fgene.2022.990849 (PMC9616467; doi:10.3389/fgene.2022.990849)
Supplement: Supplementary file 1 [file Table1.docx]

| **Table S1.** | | | | | |
| --- | --- | --- | --- | --- | --- |
| **S. No** | **Internal reference** | **Accession number** | **Primers (F/R)** | **Amplicon length (bp)** | **Tm**  **(°C)** |
| 1 | 18S ribosomal RNA gene (18S rRNA) | AF173612.1 | 5’-GAACGAGGAATTCCCAGTAAGT-3’ | 102 | 62 |
|  |  |  | 5’-ACTAAACCATCCAATCGGTAGTAG-3’ |  | 62 |
| 2 | Actin beta (ACTβ) | NM_205518.1 | 5’-TCCCTGGAGAAGAGCTATGAA-3’ | 113 | 62 |
|  |  |  | 5’-CAGGACTCCATACCCAAGAAAG-3’ |  | 62 |
| 3 | Albumin (ALB) | NM_205261.2 | 5’-TCCTGATCCGCTACACTAAGA-3’ | 98 | 62 |
|  |  |  | 5’-CTGGCAGCACTTAGTACCAATA-3’ |  | 62 |
| 4 | Beta-2 microglobulin (Beta-2MG) | Z48922.1 | 5’-ACCTCTGTCTTTCGGCTTTG-3’ | 112 | 62 |
|  |  |  | 5’-CAGAGCCTGCTTTCAGGTATT-3’ |  | 62 |
| 5 | DnaJ heat shock protein family (Hsp40) member C24 (DNAJC24) | XM_015286159.2 | 5’-TGCAGCAGCGTGAAGATAA-3’ | 140 | 62 |
|  |  |  | 5’-GCTCTTGGAGACAGCATAACA-3’ |  | 62 |
| 6 | Eukaryotic translation elongation factor 1 alpha 1 (EEF1A1) | NM_204157.2 | 5’-GCCCGAAGTTCCTGAAATCT-3’ | 102 | 62 |
|  |  |  | 5’-AACGACCCAGAGGAGGATAA-3’ |  | 62 |
| 7 | Glyceraldehyde-3-phosphate dehydrogenase (GAPDH) | NM_204305.1 | 5’-ATGGGAAGCTTACTGGAATGG-3’ | 97 | 62 |
|  |  |  | 5’-TCATCATACTTGGCTGGTTTCT-3’ |  | 62 |
| 8 | Glucuronidase beta (GUSB) | NM_001039316.2 | 5’-CCAGGCCATCTGGAAGTTATT-3’ | 105 | 62 |
|  |  |  | 5’-CGAGTCAGCTCCATATTCACTC-3’ |  | 62 |
| 9 | Hydroxymethylbilane synthase (HMBS) | XM_417846.6 | 5’-AGCCCTGAAGATTGTCTGTATG-3’ | 98 | 62 |
|  |  |  | 5’-TGCAGGGCAGATACCATATTC-3’ |  | 62 |
| 10 | Heat shock protein 10 (HSP10) | AF031309.1 | 5’-CGTAACCAAAGGAGGCATCA-3’ | 115 | 62 |
|  |  |  | 5’-CACTGGATGAATCTCACCATCC-3’ |  | 62 |
| 11 | Heat shock protein 70 (HSP70) | EU747335.1 | 5’-CAATGACAAGGGTCGCCTTA-3’ | 95 | 62 |
|  |  |  | 5’-CCCTATCTCTGTTGGCTTCATC-3’ |  | 62 |
| 12 | L-lactate dehydrogenase B chain (L-LDHB) | NP_989508.1 | 5’-TGGCAGCTTGTTCCTTCA-3’ | 98 | 62 |
|  |  |  | 5’-GACGAACACCTGCAGTTACTA-3’ |  | 62 |
| 13 | Mitochondrial ribosomal protein S27 (MRPS27) | XM_424803.6 | 5’-GCTCCCAGCTCTATGGTTATG-3’ | 124 | 62 |
|  |  |  | 5’-ATCACCTGCAAGGCTCTATTT-3’ |  | 62 |
| 14 | Mitochondrial ribosomal protein S30 (MRPS30) | NM_204939.1 | 5’-GATCCTGTGCGATTTCAGATAGA-3’ | 104 | 62 |
|  |  |  | 5’-GGAACATCTCCAAGGTCTGATT-3’ |  | 62 |
| 15 | Phosphoglycerate kinase 2 (PGK2) | NM_204985.2 | 5’-CAAGTTCTCCAAAGGAACCAAAG-3’ | 93 | 62 |
|  |  |  | 5’-GCAGTATCTCCACCACCAATAA-3’ |  | 62 |
| 16 | Protein phosphatase 2 catalytic subunit beta (PPP2CB) | NM_205124.1 | 5’-CTGTTATGGTCGGATCCAGATG-3’ | 114 | 62 |
|  |  |  | 5’-TGTAAGACCGTTGGCATGATTA-3’ |  | 62 |
| 17 | Ribosomal protein L5 (RPL5) | NM_204581.4 | 5’-AATATAACGCCTGATGGGATGG-3’ | 99 | 62 |
|  |  |  | 5’-CTTGACTTCTCTCTTGGGTTTCT-3’ |  | 62 |
| 18 | Ribosomal protein L13 (RPL13) | NM_204999.1 | 5’-GGAGTATCGCTCCAAGCTTATC-3’ | 126 | 62 |
|  |  |  | 5’-ACGTTCCTGATCGGCATAAC-3’ |  | 62 |
| 19 | Ribosomal protein L14 (RPL14) | AB046393.1 | 5’-TGCAGCTGACTGACTTTGT-3’ | 100 | 62 |
|  |  |  | 5’-GCTGCCCACTTCTCATTTATATTC-3’ |  | 62 |
| 20 | Ribosomal protein L19 (RPL19) | NM_001030929.1 | 5’-AGATACAGGGAGTCCAAGAAGA-3’ | 110 | 62 |
|  |  |  | 5’-TTGTGGATGTGCTCCATCAG-3’ |  | 62 |
| 21 | Ribosomal protein L23 (RPL23) | NM_001321599.1 | 5’-CAGCGGAAATCGTACAGAAGA-3’ | 104 | 62 |
|  |  |  | 5’-GTGATGGCTGAACCTTTCATTTC-3’ |  | 62 |
| 22 | Succinate dehydrogenase complex flavoprotein subunit A (SDHA) | NM_001277398.1 | 5’-TTGATGTCACTAAGGAGCCTATTC-3’ | 120 | 62 |
|  |  |  | 5’-GTACCACCTTATCCTCACCATTC-3’ |  | 62 |
| 23 | TATA-box binding protein (TBP) | NM_205103.1 | 5’-CCACGTACTACTGCGCTTATATT-3’ | 109 | 62 |
|  |  |  | 5’-TCTGAACAACTCTTGCGTACTT-3’ |  | 62 |
| 24 | Transferrin receptor (TFRC) | X55348.1 | 5’-GAGCAAGCCATGTCAAGATTTC-3’ | 122 | 62 |
|  |  |  | 5’-GTCTGGGCCAAGTCTGTTATAG-3’ |  | 62 |
