# Supplementary material for: Embryonic transcriptome unravels mechanisms and pathways underlying embryonic development with respect to muscle growth, egg production, and plumage formation in native and broiler chickens
Source: Front Genet. 2022 Oct 14;13:990849. doi: 10.3389/fgene.2022.990849 (PMC9616467; doi:10.3389/fgene.2022.990849)
Supplement: Supplementary file 3 [file Table2.docx]

**Table S2.**

| **S. No** | **Gene Name** | **Accession No.** | **Microarray expression** | | | | **qRT-PCR expression** | | | |
| --- | --- | --- | --- | --- | --- | --- | --- | --- | --- | --- |
|  |  |  | **7EB** | | **18TM** | | **7EB** | | **18TM** | |
| **Relative expression of DEGs that are involved in muscle development, myostatin signaling, muscle metabolism (energy sensing and storage), and protein synthesis** | | | | | | | | | | |
|  |  |  | **FC** | **P value** | **FC** | **P value** | **FC** | **P value** | **FC** | **P value** |
| 1 | MSTN | NM_001001461.1 | -1.52 | 0.07 |  |  | 0.21 | 0.3 | -2.66 | *0.08 |
| 2 | FST | NM_204638 | 2.37 | 0.13 |  |  | 1.05 | 0.11 | -0.47 | *0.04 |
| 3 | ACVR2A | NM_205367.1 |  |  |  |  | 0.56 | 0.21 | -0.01 | 0.49 |
| 4 | ACVR2B | NM_204317.1 |  |  |  |  | 0.1 | 0.41 | -0.52 | *0.01 |
| 5 | ACVR1B | XM_001231300 | 4.50 | 0.08 |  |  | -0.14 | 0.34 | -0.76 | 0.11 |
| 6 | TGFBR1 | NM_204246.1 | -2.28 | *0.01 |  |  | -0.07 | 0.43 | -0.99 | 0.06 |
| 7 | SMAD2Z | NM_204561.1 |  |  |  |  | 0.17 | 0.35 | -0.65 | 0.06 |
| 8 | MYOG | NM_204184.1 |  |  |  |  | 0.29 | 0.24 | -0.95 | 0.2 |
| 9 | MUSTN1 | NM_213580 |  |  | 1.95 | 0.09 | 0.58 | 0.17 | 1.77 | *0.04 |
| 10 | MZ2 | BX930590 | -2.27 | 0.06 | 2.45 | 0.06 | 0.04 | *0.04 | 0.03 | 0.13 |
| 11 | MTOR | XM_417614.5 |  |  |  |  | 0.01 | 0.49 | 0.56 | 0.3 |
| 12 | RPS6KA1 | NM_001109771.2 | 2.59 | 0.11 | 1.20 | *0.01 | 0.27 | 0.23 | -0.41 | 0.08 |
| 13 | CAV1 | NM_001105664 | -1.28 | *0.01 |  |  | 2.42 | *0.01 | -1.15 | *0.04 |
| 14 | CAV2 | NM_001007086 | -2.24 | *0.04 |  |  | 2.7 | *0.01 | -0.78 | *0.03 |
| 15 | CAV3 | NM_204370.2 | -3.86 | *0.03 |  |  | 0.95 | 0.07 | -0.53 | *0.03 |
| 16 | TNNT1 | NM_205114 | 2.20 | *0.05 | 2.26 | *0.04 | 0.6 | 0.21 | 0.71 | 0.19 |
| 17 | TNNI1 | BX931462 |  |  | 2.75 | *0.02 | 0.8 | 0.06 | 0.08 | 0.42 |
| 18 | TNNT3 | NM_204922 | 2.21 | *0.01 |  |  | 1.29 | 0.09 | 0.72 | 0.12 |
| 19 | CKM | NM_205507.1 | 1.71 | *0.03 |  |  | 0.03 | 0.47 | -0.24 | 0.24 |
| 20 | MAP2K6 | XM_003642348.2 |  |  |  |  | 8.32 | *0.01 | -0.28 | 0.22 |
| 21 | MAP3K7 | BX931389 | 1.76 | 0.16 |  |  | 0.13 | 0.31 | -0.33 | 0.16 |
| 22 | MAPK8IP3 | XM_424591 | 3.33 | *0.03 |  |  | 0.35 | 0.25 | -1.02 | *0.02 |
| 23 | MAPKKK4 | CR523470 | 3.48 | 0.12 |  |  | 1.27 | 0.11 | 4.21 | *0.04 |
| 24 | MAPKAPK3 | XM_414262 | 3.30 | *0.04 | 2.20 | 0.13 | -0.69 | *0.03 | -3.13 | 0.06 |
| **Relative expression of DEGs that are involved in embryo development** | | | | | | | | | | |
| 25 | HP29 | AW198329.1 | -3.36 | 0.08 | -7.56 | 0.07 | -0.12 | 0.41 | -0.61 | *0.01 |
| 26 | TOLLIP | NM_001006471.1 | 1.60 | *0.05 | 6.66 | *0.001 | 1.22 | 0.15 | -0.82 | *0.02 |
| 27 | Chain A FAS | BX935039.1 | -1.57 | 0.11 | -9.19 | *0.05 | 1.83 | 0.45 | -0.87 | *0.001 |
| 28 | D alpha | CR733292.1 | 7.32 | *0.001 |  |  | -0.26 | 0.06 | -0.29 | 0.18 |
| 29 | UP5 | ES605836.1 | 6.62 | 0.10 |  |  | 1.83 | 0.45 | -0.87 | *0.001 |
| 30 | Asw | CN225783.1 | -1.57 | 0.11 | -9.19 | *0.05 | -0.11 | *0.01 | 1178.4 | *0.04 |
| 31 | CREM | NM_204387 | -3.04 | *0.01 |  |  | 0.87 | *0.04 | -2.28 | *0.05 |
| 32 | MYBPC1 | BX935207 | 1.90 | *0.01 |  |  | 0.88 | 0.14 | 5.38 | *0.004 |
| 33 | MYBPC2 | NM_001044659 | 2.04 | *0.02 |  |  | 0.2 | 0.32 | -1.91 | *0.04 |
| 34 | RQCD1 | NM_001006521 | -1.47 | 0.14 |  |  | 2.76 | *0.02 | -0.4 | 0.17 |
| 35 | LMOD3 | BX935813 | 1.73 | 0.06 |  |  | 0.35 | 0.23 | -1.06 | *0.04 |
| 36 | DKK2 | XM_420494 | 3.13 | *0.04 |  |  | 0.94 | 0.1 | -1.41 | *0.04 |
| 37 | CD9 | NM_204762 | -4.58 | *0.0001 |  |  | 0.79 | 0.14 | 1.15 | *0.03 |
| 38 | PLIN1 | NM_001127439 | 2.26 | *0.05 |  |  | 2.12 | 0.06 | -4.52 | *0.05 |
| 39 | PLN | NM_205410 | -4.41 | 0.12 | 4.39 | *0.03 | 6.14 | *0.03 | -2.72 | *0.04 |
| 40 | GPM6A | NM_001012579 | -4.69 | *0.03 |  |  | 0.32 | 0.16 | 0.08 | 0.46 |
| 41 | GPM6B | NM_001012545 | -3.21 | *0.003 |  |  | 1.92 | *0.05 | -0.4 | *0.03 |
| 42 | AGTR1 | NM_205157 | -4.09 | 0.07 |  |  | 0.49 | -2.1 | 0.2 | 0.06 |
| 43 | AGTRAP | BX930324 | -2.03 | *0.04 |  |  | -0.01 | 0.49 | -1.06 | *0.03 |
| 44 | FSTL1 | NM_204638 | 2.59 | 0.18 |  |  | 0.77 | 2.42 | 0.2 | 0.08 |
| 45 | FGCP | BG642009.1 | -4.49 | *0.01 | -7.25 | 0.11 | 0.09 | 0.42 | 45.68 | *0.004 |
| 46 | WAPFK | NP9672441 |  |  | -1.14 | 0.12 | 0.43 | 0.13 | -1.11 | *0.01 |
| 47 | GPI | NM_001006128 | -1.87 | *0.04 |  |  | 1.38 | 0.09 | 1.63 | *0.02 |
| **Relative expression of DEGs that are involved in fatty acid metabolism** | | | | | | | | | | |
| 48 | CPT1 | DQ314726.1/ENSGALT00000011466 | 1.79 | 0.16 |  |  | 0.3 | 0.18 | -0.9 | *0.0002 |
| 49 | ACSL1 | NM_001012578.1 | -1.09 | *0.01 | 2.12 | *0.02 | 0.4 | 0.13 | -1.1 | *0.05 |
| 50 | ECHS1 | NM_001277395.1/CR407482 |  |  | 1.75 | 0.13 | 0.6 | 0.09 | -1.4 | *0.007 |
| 51 | HADH | NM_001277897.1/NM_205056 |  |  | 2.29 | 0.08 | 0.6 | 0.3 | -1.5 | *0.05 |
| 52 | SREBP1 | AJ310768.1 |  |  |  |  | -0.1 | 0.35 | -1.2 | *0.001 |
| 53 | FGFR1 | NM_205510.1 | 1.17 | *0.05 |  |  | 0.8 | 0.12 | -1.6 | *0.04 |
| 54 | FOXO3 | MK861853.1/XM_001234495 | 1.92 | 0.17 |  |  | 0.3 | 0.25 | -1.5 | *0.01 |
| 55 | ACACA | NM_205505.1 | 2.79 | 0.11 |  |  | 2 | 0.19 | -2.9 | *0.03 |
| 56 | ACACB | XM_025155692.1/XM_428114 | 2.32 | 0.11 |  |  | 0.2 | 0.3 | -1.5 | *0.02 |
| 57 | PPARγ | AF163811.1/XM_414479 | 1.51 | 0.13 | 2.25 | *0.01 | 1.3 | *0.03 | -0.8 | *0.02 |
| 58 | MYF6 | FJ882409.1/NM_001030746 | 1.44 | *0.04 |  |  | 1.8 | *0.04 | -0.6 | *0.04 |
| 59 | FGF2 | NM_205433.1 |  |  |  |  | 0.6 | 0.2 | -1.1 | *0.002 |
| 60 | DNMT3A | NM_001024832.1 | 1.90 | *0.03 |  |  | 1.1 | 0.09 | -1.2 | *0.003 |
| 61 | ACOX2 | XM_015293306.2/XM_414406 | -4.18 | *0.04 |  |  | 2.4 | *0.04 |  |  |
| 62 | FASN | NM_205155.3 | 1.44 | 0.26 |  |  | 82 | *0.04 |  |  |
| **Relative expression of DEGs that are involved in cell signaling and egg production** | | | | | | | | | | |
| 63 | IGF1R | NM_205032 | 2.70 | 0.38 |  |  | 0.48 | 0.25 | -0.88 | 0.06 |
| 64 | IGFBP1 | NM_001001294 | -3.29 | *0.01 |  |  | 0.08 | 0.41 | -2.33 | *0.06 |
| 65 | PRKAA1 | NM_001039603.1 |  |  |  |  | 0.03 | 0.47 | -1.1 | *0.02 |
| 66 | PRKAA2 | NM_001039605.1 | 1.42 | 0.35 |  |  | -0.02 | 0.48 | -1 | *0.05 |
| 67 | SLC2A3 | NM_205511 | -2.84 | *0.03 |  |  | 0.4 | 0.21 | -1.14 | *0.05 |
| 68 | JAK1 | NM_204870 | -1.61 | *0.01 |  |  | 0.46 | 0.14 | -0.91 | *0.005 |
| 69 | JAK2 | NM_001030538 | 4.17 | *0.03 | 1.23 | *0.03 | 0.95 | *0.02 | 0.11 | 0.35 |
| 70 | JAKMIP2 | CR390426 | 2.37 | *0.01 |  |  | 1.23 | *0.001 | -0.18 | 0.28 |
| 71 | JAKMIP3 | XM_426548 | 3.90 | *0.01 |  |  | 0.56 | 0.12 | -1.93 | *0.05 |
| 72 | HSTF 1 | BM440477 | 2.79 | 0.16 |  |  | 0.7 | 0.1 | 10.3 | *0.001 |
| 73 | HSPA8 | NM_205003 | -2.12 | *0.03 |  |  | 0.26 | 0.21 | -1.66 | *0.05 |
| 74 | HSBP1 | NM_001112809 | -2.03 | *0.04 |  |  | 0.87 | 0.21 | -3.11 | 0.17 |
| 75 | HSP70 | AJ301880 | 6.23 | 0.30 | 4.84 | *0.001 | 2.4 | *0.02 | 0.52 | 0.09 |
| 76 | HBC9 | BX950823 |  |  | 3.92 | 0.16 | 0.21 | 0.29 | -1.08 | 0.07 |
| 77 | HSPD1 | NM_001012916 | -2.18 | *0.01 |  |  | 1.36 | 0.07 | -0.93 | *0.05 |
| 78 | HSPA5 | NM_205491 | -2.15 | *0.02 |  |  | 0.7 | 0.09 | -1.42 | *0.04 |
| 79 | ALPBP | NM_001044633.1 | -3.82 | *0.01 | -7.06 | 0.11 | 0.75 | 0.15 | -1.09 | 0.07 |
| 80 | MHCRCP2B | CR406681.1 |  |  | -7.00 | 0.13 | 0.3 | 0.2 | -0.76 | *0.05 |
| 81 | AARIB | XM_001231300 | 4.50 | 0.08 |  |  | 0.69 | 0.06 | -0.33 | 0.18 |

**FC:** Fold change; ***** P≤0.05; Highlighted **red** and **green** color indicates up and down-regulation, respectively; Gray color indicates genes not differentially regulated.
